# High-throughput phenotypic analysis of plant and curd growth dynamics during the whole growth period of cauliflower based on instance segmentation

**DOI:** 10.3389/fpls.2026.1836813

**Published:** 2026-05-13

**Authors:** Saichuan Cheng, Sidi Wu, Qingqing Shao, Mindong Chen, Jianting Liu, Boyin Qiu, Haisheng Zhu

**Affiliations:** 1 Fujian Key Laboratory of Vegetable Genetics and Breeding, Crops Research Institute, Fujian Academy of Agricultural Sciences, Fuzhou, China; 2 School of Mathematics and Statistics, Fuzhou University, Fuzhou, China

**Keywords:** Cauliflower, deep learning, growth dynamics, instance segmentation, time series

## Abstract

Efficient phenotyping monitoring of cauliflower is crucial for its breeding and production. However, traditional manual measurement methods are time-consuming and labor-intensive, and existing deep learning (DL) methods mostly focus on the seedling stage, lacking systematic research covering the entire growth period. In this study, RGB images of cauliflower from seedling to harvest were collected. Through systematic screening and evaluation of instance segmentation models, accurate segmentation of plants and curds was achieved, and plant canopy width, leaf area, and curd traits were automatically extracted to track their dynamic changes. Evaluation results showed that YOLO12s-seg was the optimal model. It can achieved a segmentation mask mAP_50_ of 99.4% for plants and curds in sparsely planted images and showed an advantage in identifying partially occluded early curds beneath inner leaves. Traits such as plant canopy width and curd diameter automatically extracted from segmentation results were highly consistent with manual measurements (R^2^ > 0.90). Furthermore, the Richards model and Sine model were used to accurately fit the growth dynamics of leaf area and curd area, respectively. Based on growth kinetics, curds were classified into three types: mature and compact type, peak-burst type, and steady-increase type. Cluster analysis of 47 germplasms based on high-throughput phenotyping data revealed four groups and their growth characteristics: comprehensively coordinated type, mid-maturity compact type, large high-yield type, and curd-dominant type. Integrating the above functions, a platform for cauliflower growth monitoring and phenotypic analysis was developed. It provided full-process support from automatic image processing to growth dynamic analysis. This work provides an effective automated solution for high-throughput phenotyping analysis and growth dynamic monitoring of cauliflower, and offers a referable analytical framework for crop growth pattern research and intelligent breeding decision-making.

## 1 Introduction

With the development of agricultural digital transformation and intelligent systems, the efficient and accurate acquisition of crop phenotypic traits has become a key issue in plant science and precision agriculture (Zhang et al., 2019). Traditional manual or semi-automatic measurement methods are inefficient and highly subjective, making it difficult to meet the demand for high-throughput phenotypic data in modern breeding and cultivation management (Pan et al., 2022). In recent years, DL technologies, especially convolutional neural networks (CNNs) and their architectures combined with Transformers, have shown strong potential in agricultural image recognition, segmentation, and classification tasks, providing a technical foundation for the dynamic and non-destructive monitoring of crop growth processes.

Cauliflower (*Brassica oleracea* var. *botrytis* L.) is a nutritious cruciferous crop (Zhang et al., 2025a) with anti-cancer and cancer-preventive effects (Melim et al., 2022). Its market demand is increasing, and its phenotypic traits such as leaf area, plant canopy width, and curd size are directly related to photosynthetic efficiency, biomass accumulation, and final yield (Richards, 2000; Kruger and Volin, 2006). Traditional measurement methods are difficult to achieve continuous tracking throughout its whole growth period. Therefore, the automatic and high-throughput extraction of key traits of cauliflower using computer vision technology is of great significance for variety breeding and cultivation optimization.

Compared with seedling-stage phenotyping or single-time-point trait extraction in other crops, whole-growth-period phenotypic monitoring in cauliflower involves several specific technical challenges. First, plant architecture changes substantially across developmental stages, from sparse seedlings to overlapping rosette leaves and finally to curd formation, resulting in strong intra-class variation in shape and scale. Second, curds emerge within the canopy and are often partially obscured by inner leaves, which makes accurate boundary delineation difficult in RGB images. Third, changes in camera height across growth stages complicate scale calibration and pixel-to-area conversion. Fourth, developmental timing differs markedly among germplasms, making it difficult to compare varieties using observations from a single calendar date. These factors limit the direct applicability of methods developed only for early growth stages or for crops with more exposed reproductive organs.

At present, object detection and instance segmentation models based on DL, especially CNNs, have been widely applied in continuous time-series phenotypic monitoring of various crops (Mohanty et al., 2016; Stavness, 2017). The focus is shifting from static trait acquisition at a single time point to in-depth analysis of growth dynamics.

Beyond single time-point measurements, dynamic phenotypic traits provide additional biological and practical value. Time-series changes in leaf expansion and curd development can reveal growth rhythm, developmental transitions, source–sink coordination, and variation in effective curd expansion duration that cannot be captured by final organ size alone. For breeding, such dynamic descriptors help distinguish germplasms with different growth strategies, maturity patterns, and harvest potential. For crop management, they can support earlier prediction of curd developmental transition and more precise determination of the optimal harvest window.

The model architectures adopted in these studies are diverse, ranging from classic CNNs to emerging Transformer models (Thakur et al., 2025).

For multi-stage dynamic monitoring covering the whole growth period, relevant studies have established a complete process from recognition to analysis. For example, Du et al. (2020) constructed a multi-stage model of “object detection-semantic segmentation-phenotypic analysis” based on CNNs to conduct high-throughput dynamic monitoring of lettuce during the entire growth period, successfully extracting static traits and generating growth curves. Hou et al. (2024) collected images of four lettuce varieties across the whole growth period and improved the Mask R-CNN model (replacing the backbone with RepVGG and adding a phenotypic branch) to achieve end-to-end accurate estimation of five key traits including fresh weight and plant height. However, although such studies have collected time-series data, in-depth mining of growth dynamic patterns remains insufficient.

In terms of high-precision segmentation and recognition for specific growth stages, numerous studies have improved performance by optimizing model structures. At the seedling stage, Wang et al. (2023) improved the YOLACT model and proposed the YOLACT-RFX algorithm, achieving high-precision segmentation and seedling-stage recognition of cabbage seedlings, with a mAP_50_ of 84.4%. Xu et al. (2023) proposed an automatic counting method based on semi-supervised learning and UAV imagery for the dynamic monitoring of leaf number in maize seedlings. This method used the Noisy Student framework to co-train the SOLOv2 model (for seedling segmentation) and YOLOv5x model (for leaf counting), enabling efficient learning with a small amount of annotated data and effectively monitoring the leaf growth process. Similarly, Jiang et al. (2024) established the Seg-FL model based on YOLOv5s-seg for dynamic monitoring and grading of luffa seedlings, with a mAP_50_ of 97.03%. Cao et al. (2025) optimized the YOLOv11-HSECal model for the vigor evaluation of okra seedlings under salt stress, achieving a good balance between accuracy and efficiency. These studies indicate that model optimization for specific stages and tasks can effectively improve the accuracy of trait extraction. However, these studies mainly target early growth stages or task-specific scenarios, and their methodological assumptions may not transfer well to cauliflower across the whole growth period. In particular, the emergence of curds inside dense inner leaves and the strong temporal heterogeneity among germplasms make full-cycle phenotyping of cauliflower more challenging than seedling-stage monitoring alone.

In using multi-source data and complex models to deal with complex field scenarios, studies have demonstrated the adaptability of different technical paths. Psiroukis et al. (2022) combined UAV remote sensing with Faster R-CNN and CenterNet models, realizing for the first time the extraction and change monitoring of multi-grade maturity traits of broccoli curds during the entire maturation period. To monitor early maize growth, Stypułkowska and Rokita (2024) simultaneously collected RGB and multispectral data and compared the HTC series and Mask2Former models. They found that different models had different adaptability to data sources: HTC performed better on multispectral data, while Mask2Former achieved the highest recognition accuracy on RGB images.

Regarding the development trend and application selection of model architectures, a notable phenomenon is that although new architectures such as Vision Transformer show advantages in complex scenarios, classic CNN architectures (such as the YOLO series) optimized for specific agricultural phenotypic tasks often achieve a more practical balance between accuracy and efficiency. For example, in tasks such as leaf area estimation or seedling vigor evaluation, improved lightweight YOLO models (e.g., YOLOv11-AreaNet, YOLOv11-HSECal) (Cao et al., 2025; Zhang et al., 2025b) may outperform general Transformer-based segmentation models. More importantly, for most researchers aiming to solve practical breeding problems, the significance of technological trends lies in providing better tool choices rather than having to experience the entire process of model improvement. Moreover, mainstream object detection and segmentation frameworks are actively absorbing and integrating new architectural ideas including attention mechanisms to maintain competitiveness during rapid iterations. For instance, latest versions such as YOLO12s-seg have integrated self-attention or convolutional block attention modules (CBAM) from Transformers into their network design (Tian et al., 2025), becoming “trend-compliant ready-to-use models”. This reflects a pragmatic trend: for most breeding applications, directly screening and utilizing mainstream frameworks as off-the-shelf tools and focusing core efforts on phenotypic data mining and genetic analysis may become a more efficient research paradigm.

In summary, although existing studies have applied diverse models (such as Mask R-CNN, YOLACT, SOLOv2, YOLO series, Mask2Former, etc.) to achieve progress in trait monitoring of different crops at different time series (Kamilaris and Prenafeta-Boldú, 2018), obvious limitations still exist. First of all, most studies focus on the seedling stage, and systematic monitoring of the whole growth period (especially the maturation stage) of crops such as cauliflower is scarce; second, research focuses mostly on the optimization of model structure itself, with insufficient in-depth mining of time-series data obtained from segmentation and analysis of growth laws; finally, for the important crop cauliflower, a complete technical scheme that can cover the whole growth period and realize dynamic extraction and analysis of multiple key traits has not yet been established.

To address the above problems, this study takes multi-variety cauliflower as the research object and carries out whole-growth-period monitoring from the seedling stage, rosette stage to commercial harvest stage. By collecting RGB images of cauliflower at different periods, a high-quality annotated dataset for instance segmentation is constructed. Multiple mainstream instance segmentation models are systematically screened and evaluated, and the most suitable model for cauliflower image segmentation is selected to achieve accurate segmentation of plants and curds. Based on the segmentation results, key traits such as plant canopy width, leaf area, curd vertical and horizontal diameter, and curd area are automatically extracted, and their dynamic change laws are tracked. Finally, an estimation method for cauliflower growth period and commercial harvest time is established. This study aims to provide efficient and reliable technical support for cauliflower growth monitoring, phenotypic analysis, and variety breeding, and the methodological framework can also provide a reference for whole-growth-period phenotypic research of other crops.

## 2 Materials and methods

### 2.1 Experimental materials and image acquisition

In this experiment, 47 autumn-ecotype inbred lines of cauliflower were used for image collection, provided by the Crop Research Institute, Fujian Academy of Agricultural Sciences. The materials were planted in October 2024 in a greenhouse of the Crop Research Institute, Fujian Academy of Agricultural Sciences (119.34° E, 26.13° N), with a plant spacing of 1 meter.

**Figure 1:**
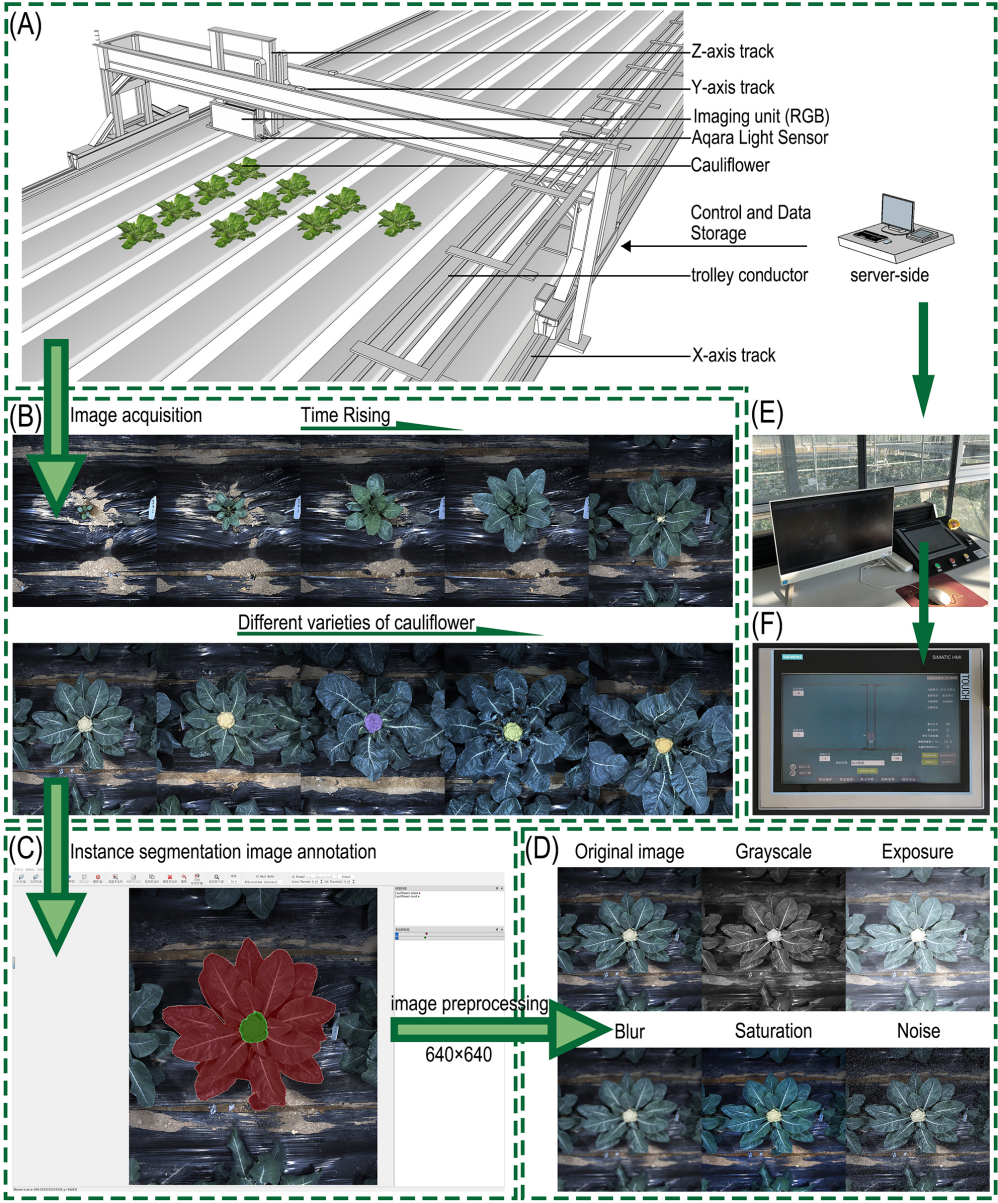
Whole-time-series image acquisition and dataset construction: **(A)** High-throughput field crop phenotyping detection system; **(B)** Time-series images of cauliflower plants; **(C)** Image annotation of cauliflower plants and curds; **(D)** Image data augmentation; **(E)** Server side; **(F)** Acquisition control panel.

RGB images were acquired using a high-throughput field crop phenotyping system developed by Wuhan Gufeng Optoelectronic Technology Co., Ltd. (**Figure 1A**). In the early growth stage, the camera height was set at 1.5 m to capture more seedling details, and raised to 2.2 m after 70 days. Imaging started on the 1st day after transplanting at an interval of 3 days. Once curds appeared on the first cauliflower plant, the interval was changed to 1 day, and imaging ended when the curds of the last germplasm matured. Image files were named with timestamps in the format “X-Y-YYYY-MM-DD-number”. A total of 7917 valid images were collected at the seedling, rosette, and commercial harvest stages. All images had a resolution of 2096×2508 pixels.

### 2.2 Dataset construction

To ensure that the selected image samples covered different developmental stages including the seedling, rosette, and commercial harvest stages, images from the 1st day after transplanting and every subsequent 6 days were selected from all collected samples for analysis. Finally, a total of 3252 images were annotated.

The 6-day sampling interval was adopted to balance annotation workload, temporal coverage, and morphological diversity. Because the full dataset contained 7,917 images and temporally adjacent images were often highly similar, annotating all images would have introduced substantial redundancy while greatly increasing annotation cost. Selecting images at 6-day intervals allowed representative coverage of the seedling, rosette, and curd development stages while preserving sufficient morphological variation for model training.

Labelme software was used for dataset annotation, following the YOLO dataset format. Plants were labeled as “Cauliflower plant”, and curds were labeled as “cauliflower curd” (**Figure 1C**).

Meanwhile, to meet the training requirements of instance segmentation models, the JSON files generated from annotation were standardized. Key parameters such as image resolution, class identifiers, and bounding box coordinates were extracted via scripts, and pixel coordinates were normalized and converted into a model-readable TXT format. To further improve the robustness of the model in complex real-world environments (Tang et al., 2025), various preprocessing and data augmentation operations were performed on images before training, mainly including grayscale conversion, simulated overexposure, Gaussian blur, saturation adjustment, and random noise addition (**Figure 1D**). These processes were designed to simulate common disturbances in field imaging, such as uneven illumination, camera shaking, focusing deviation, weather changes, and sensor noise. To avoid potential temporal data leakage, the dataset was split at the individual-plant level rather than the image level. That is, all images belonging to the same plant across different acquisition dates were assigned to only one subset. The final ratio of training, validation, and test sets was 7:2:1. This strategy ensured that model evaluation was performed on plants that were not seen during training.

### 2.3 Instance segmentation models

To systematically evaluate the performance of instance segmentation models for the recognition and segmentation of cauliflower plants and curds in time-series images, eight advanced models with distinct architectural characteristics were selected for comparative analysis in this study, covering both classic methods and recent advances to comprehensively investigate their applicability under scenarios of varying complexity.

The selected models included Mask R-CNN (a classical two-stage baseline model), HTC (a hybrid task cascade framework with strong performance in complex boundary processing), SOLOv2 (an anchor-free single-stage model based on location awareness), YOLACT (a real-time single-stage model based on prototype mask generation), Mask2Former (a universal segmentation architecture based on Swin Transformer and attention mechanisms), as well as YOLOv8-seg, YOLO11s-seg, and YOLO12s-seg (real-time instance segmentation series based on the CSPDarknet backbone), which achieve a favorable balance between speed and accuracy.

These models represent different technical paradigms, including two-stage and single-stage designs, CNN-based and Transformer-based backbones, and optimization directions for either real-time performance or high precision. They enable comprehensive comparison in terms of segmentation accuracy, boundary quality, inference speed, and occlusion resistance. The model structures and characteristics are detailed in **Table 1**.

**Table 1:** Comparison of instance segmentation models.

Model	Type	Backbone	Key characteristics
Mask R-CNN	Two-stage	ResNet-50	Classic baseline, high stability (He et al., 2020).
HTC	Two-stage	ResNet-50	Hybrid task cascade, good for complex boundaries (Chen et al., 2019).
SOLOv2	One-stage	ResNet-50	Anchor-free,segments by location (Location-aware) (Wang et al., 2020).
YOLACT	One-stage	ResNet-50	Prototype mask generation, prioritized speed (Bolya et al., 2019).
Mask2Former	Transformer	Swin-T	Handles occlusion well via attention mechanisms (Cheng et al., 2022).
YOLOv8s/11s/12s-seg	One-stage	CSPDarknet	State-of-the-art speed/accuracy balance (Chen et al., 2025; Sapkota and Karkee, 2025; Tian et al., 2025).

### 2.4 Model training configuration

All experiments in this study were conducted on a computer with the Windows 11 operating system. The CPU is an Intel 13th Gen Intel(R) Core(TM) i9-13900HX, and the GPU is an NVIDIA GeForce RTX 4060. The training environment was built using Anaconda3, with Python 3.11, PyTorch 2.2.2, and torchvision 0.17.2 as the main programming and DL frameworks. NVIDIA CUDA 11.2 was used to accelerate computation and reduce training time. During training, all images were uniformly resized to a resolution of 640 × 640.

Detailed training parameters of the models are listed in **Table 2**. A standardized training strategy was adopted to fully train the models and optimize their performance. The stochastic gradient descent (SGD) optimizer was used with a momentum of 0.9 and a weight decay of 0.0001 (Loshchilov and Hutter, 2019). SGD was selected for its stable convergence and well-established optimization theory. The momentum term accelerates convergence and smooths the optimization process, while weight decay applies L2 regularization to model parameters to prevent overfitting (Ruder, 2017). Training started with an initial learning rate of 0.01, followed by a linear decay strategy. The batch size was set to 16 images per batch. Training epochs were dynamically adjusted so that training terminated automatically when performance on the validation set plateaued, avoiding unnecessary computational cost.

**Table 2:** Model training parameters.

Parameters name	Parameters value
Epoch	100
Batch size	16
Image size	640×640
Optimizer	SGD
Learning Rate	0.01
Momentum	0.9
Weight Decay	0.0001

### 2.5 Trait extraction and visual image processing

#### 2.5.1 Extraction and visualization of plant canopy width, leaf area, horizontal and vertical diameters of curd, and curd area

**Figure 2:**
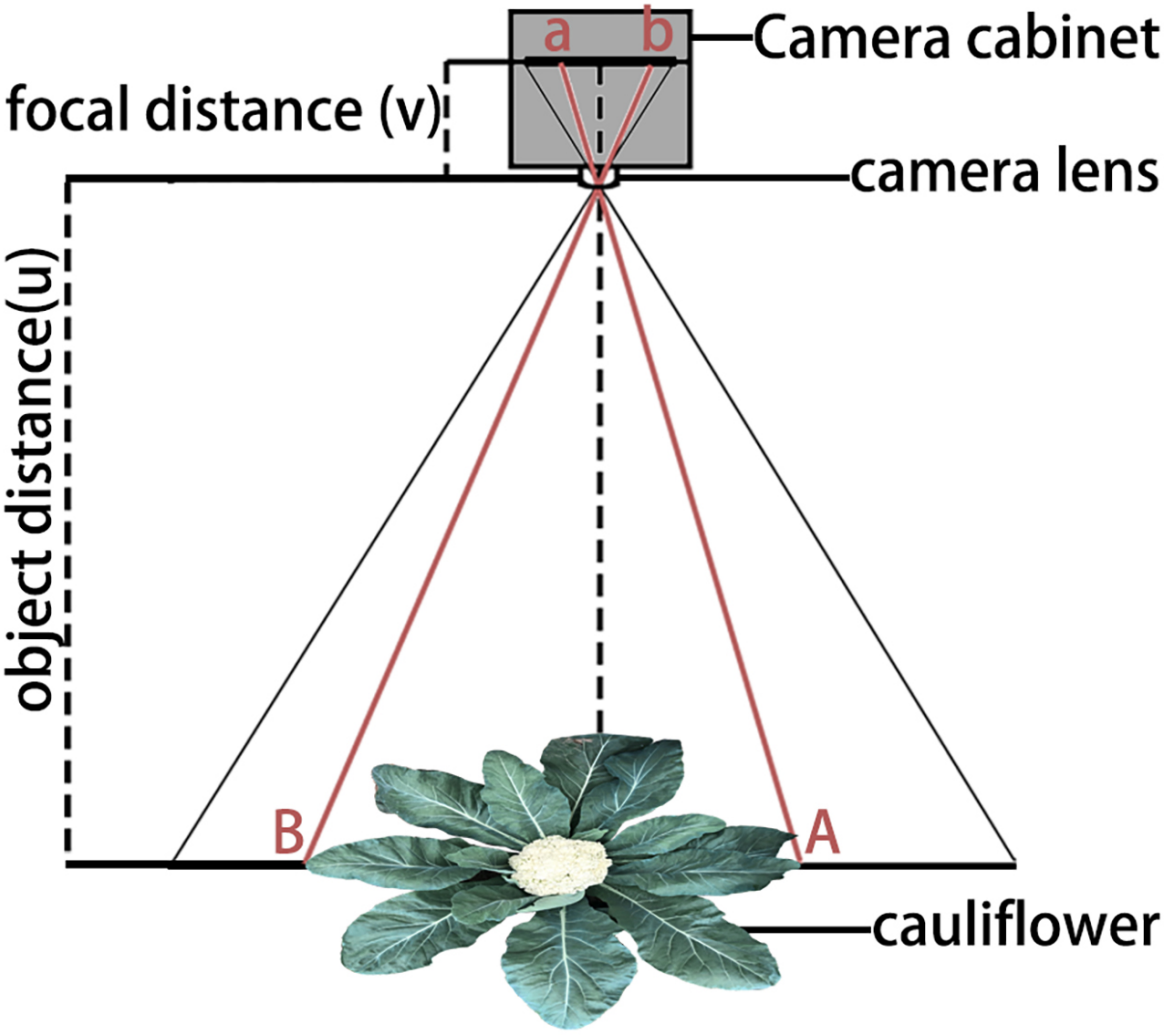
Principle of scale conversion.

The trained optimal model was used to perform instance segmentation on all 7,917 collected images. OpenCV was employed to extract the pixel areas of the segmented images of cauliflower plants and curds, as well as the annotations of trait data and shooting heights. Firstly, the pixel areas (m) were converted into the actual leaf area (LA) and curd area (CA) of the plants based on the relationship between the object distance and image distance of the camera (**Figure 2**) (Kierdorf et al., 2023). During conversion, the image acquisition height was set to 1.5 m in the early stage and adjusted to 2.2 m for images collected after 70 days.

To calibrate image scale, a reference ruler with known dimensions was placed at canopy level before image acquisition, and calibration images were collected separately at the two fixed camera heights (1.5 m and 2.2 m). Based on the imaging geometry shown in **Figure 2**, pixel-to-length conversion coefficients were determined for each height, and the corresponding pixel-to-area coefficients were derived accordingly. During phenotypic extraction, the appropriate calibration coefficient was selected according to the recorded camera height of each image. Plant canopy width and curd diameter were calculated from the detected bounding boxes, whereas leaf area and curd area were converted from segmented pixel areas to actual values using the calibrated scale factors. Manual ground-truth measurements collected at the commercial harvest stage were used to validate the accuracy of the image-derived traits.

Subsequently, the plant canopy width (PCW) and curd diameter (FCD) were obtained by conversion according to the length−to−width ratio of the detected bounding boxes. Finally, these data were clearly labeled at the corresponding positions on the images, thus achieving quantitative and visual analysis of the growth morphology of cauliflower.

The schematic diagram of the principle is shown in **Figure 2**, and the conversion formula is shown in Equation 1.

(1) $$\text{LA}=\frac{\text{u}}{\text{v}}\times \text{m}$$

where LA is the actual leaf area, m is the pixel area from instance segmentation, u is the object distance, and v is the image distance.

#### 2.5.2 Extraction of growth rates for plant canopy width, leaf area, horizontal and vertical diameters of curd, and curd area

The complete time-series dataset used in this study adopted timestamps in the format of “X-Y-YYYY-MM-DD-number”. For the extracted trait data, regular expressions were used to extract time information from these timestamps and calculate the time interval (unit: day) between consecutive frames.

The real-time growth rates of cauliflower plants and curds were calculated according to the following formula, so as to analyze the changes in growth rates of cauliflower varieties at different growth stages. The calculation formula is shown in Equation 2.

(2) $$\text{Growth Rate}=\frac{{\text{Area}}_{\text{current}}-{\text{Area}}_{\text{previous}}}{{\text{t}}_{\text{i}}-{\text{t}}_{\text{i}-1}}$$

where Growth Rate represents the growth rate of leaves or curds.

Area_current_ and Area_previous_ denote the leaf or curd area in the current frame and the previous frame, respectively.

t_i_ and t_i-1_ represent the timestamps of the current frame and the previous frame, respectively.

Notably, the time interval is not limited to consecutive frames; it can also represent any user-defined time window, allowing flexible analysis of cauliflower growth rates at different time scales.

#### 2.5.3 Trait aggregation and statistical analysis

For germplasm-level statistical analysis, phenotypic traits extracted from time-series images were first obtained for individual plants and then averaged across the three biological replicates of each germplasm. The resulting germplasm mean values (n = 47) were used for correlation analysis and clustering analysis to avoid pseudoreplication and to focus on inter-germplasm variation. Pearson’s correlation coefficients were calculated to assess pairwise relationships among traits. Prior to hierarchical clustering, all trait values were standardized by z-score normalization. Hierarchical clustering was performed using Euclidean distance and Ward’s linkage method. All statistical analyses were conducted using IBM SPSS Statistics 27.0.1.

For traits measured repeatedly over time, the values used in germplasm-level correlation and clustering analyses were defined according to their biological meaning; specifically, plant canopy width (PCW), leaf area (LA), curd area (CA), and curd diameter (FCD) refer to their final observed values, whereas growth-related variables were calculated from the corresponding time-series data.

### 2.6 Evaluation metrics for segmentation results

#### 2.6.1 Evaluation metrics for instance segmentation

Instance segmentation performance was evaluated using Precision (P), Recall (R), mean Average Precision at IoU = 0.50 (mAP_50_), and mean Average Precision over multiple IoU thresholds from 0.50 to 0.95 (mAP_50-95_). In addition, the number of parameters (Params), floating-point operations (FLOPs), and frames per second (FPS) were used to assess model complexity and inference efficiency. These metrics were used to comprehensively compare segmentation accuracy, computational cost, and real-time applicability of different models. The standard definitions and formulas of these evaluation metrics are provided in the **Appendix** (Wan et al., 2023; Khanam and Hussain, 2024).

#### 2.6.2 Regression analysis evaluation and goodness-of-fit indicators

The predicted values of leaf area and curd area obtained by conversion based on segmented pixel area and ratio need to be verified for accuracy using manually measured true values as the standard. A total of 141 plants and curds at commercial harvest stage were used as verification materials. Regression analysis was performed between the image-derived and manually measured values of plant canopy width and curd diameter, and the coefficient of determination (R^2^), root mean square error (RMSE), and mean absolute error (MAE) were calculated to verify the accuracy of the extracted phenotypic traits.

Several candidate models were fitted to the leaf area and curd area growth curves of different cauliflower germplasms. The goodness-of-fit indicators selected were the R^2^, RMSE and reduced chi-square. The calculation formulas are shown in Equations 3–6.

(3) $$R^{2}=\frac{\sum {\left(-y_{i}^{\prime }+y_{i}\right)}^{2}}{\sum {\left(-y_{i}+y_{i}^{\prime }\right)}^{2}}$$

(4) $$\text{RMSE}=\sqrt{\left(\left(1/\text{n}-\text{k}-1\right)*{\sum \left({\text{y}}_{\text{i}}^{\prime }-{\text{y}}_{\text{i}}\right)}^{2}\right)}$$

(5) $$\text{MAE}=\frac{1}{\text{n}}\sum \left|{\text{y}}_{\text{i}}^{\prime }-{\text{y}}_{\text{i}}\right|$$

(6) $${\text{χ}}^{2}=\sum_{\text{i}=1}^{\text{n}}{\left(\frac{{\text{y}}_{\text{i}}-{\text{y}}_{\text{i}}^{\prime }}{{\text{σ}}_{\text{i}}}\right)}^{2}$$

where n is the number of samples, y_i_ is the measured value, ${\text{y}}_{\text{i}}^{\prime }$ is the predicted value, k is the number of independent variables in the model, and σ_i_ is the standard error of the i-th observation.

#### 2.6.3 Software platform implementation

A software platform, BroccoPheno, was developed as a pure front-end application for cauliflower phenotypic analysis. The platform was implemented using TypeScript and React, with Vite as the development and build toolchain, and supports local execution in a Node.js environment. BroccoPheno integrates functional modules for model configuration, image-set import, time-range filtering, trait selection, analysis control, visualization, and result export. Uploaded image sets are parsed according to standardized filename rules and automatically organized by plant identity and acquisition date, thereby supporting whole-growth-period phenotypic analysis. The platform provides dynamic trait visualization, detailed tabular summaries, and growth-analysis outputs, and the final results can be exported as structured CSV files for subsequent analysis and record keeping. Graphical visualization was implemented using Recharts, and the interface was organized using reusable components and standardized client-side data-processing utilities to improve maintainability and interaction consistency. Due to the confidentiality requirements of the project, the current version of BroccoPheno is not publicly available.

## 3 Results

### 3.1 Performance evaluation and screening of instance segmentation models

#### 3.1.1 Comprehensive performance evaluation of instance segmentation models

**Figure 3:**
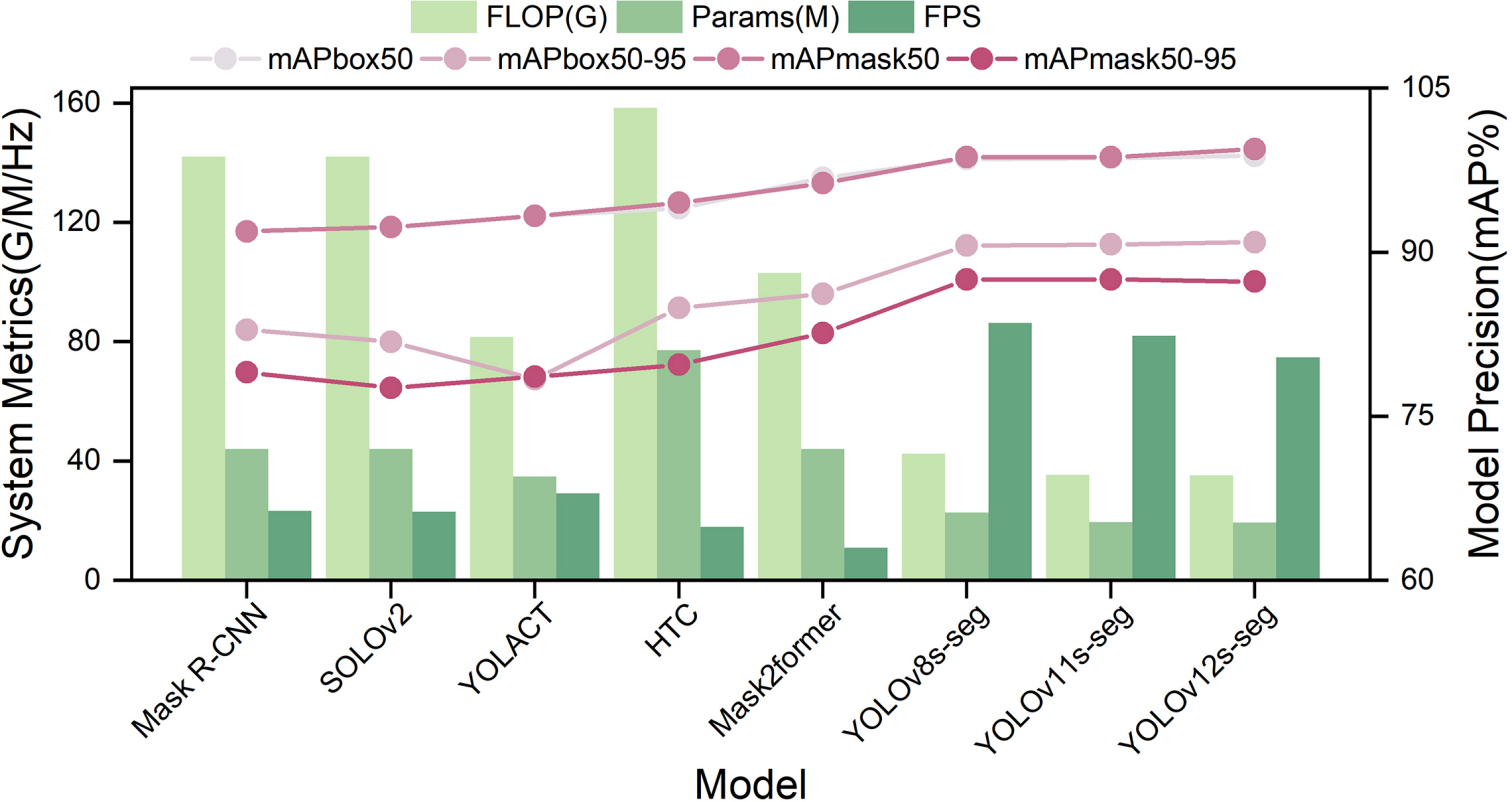
Comparative analysis of performance indicators of different models.

The model training results are shown in **Figure 3**; **Table 3**. All models exhibited extremely high overall recognition accuracy on the sparsely planted cauliflower dataset, confirming that the sparse planting strategy effectively ensured the separability of individual plants. Among them, lightweight models based on the YOLO framework performed particularly prominently: YOLO12s-seg achieved a mask segmentation accuracy (mAPmask_50_) of 99.40% and a comprehensive detection index (mAPbox_50-95_) as high as 90.90%, while maintaining only 19.3M parameters and an inference speed of 74.0 FPS. In comparison, YOLOv8s-seg achieved nearly equally excellent accuracy (mAPmask_50_ 98.70%, mAPbox_50-95_ 90.60%) with an even faster speed of 86.20 FPS. In contrast, traditional two-stage models such as HTC, although achieving 94.00% mAPbox_50_ and 84.90% mAPbox_50-95_, showed obvious disadvantages in efficiency due to their 158.30G FLOPs and only 17.80 FPS speed.

**Table 3:** Quantitative comparison of segmentation performance, model complexity, and inference efficiency of different instance segmentation models.

Model	FLOP(G)	Params(M)	FPS	mAPbox_50_	mAPbox_50-95_	mAPmask_50_	mAPmask_50-95_
Mask R-CNN	142.00	43.98	23.20	91.90	82.90	91.90	79.00
SOLOv2	142.00	43.99	23.00	92.30	81.80	92.30	77.60
YOLACT	81.54	34.73	29.10	93.30	78.40	93.30	78.60
HTC	158.30	77.16	17.80	94.00	84.90	94.50	79.70
Mask2former	103.00	44.00	10.90	96.80	86.20	96.30	82.60
YOLOv8s-seg	42.40	22.70	**86.21**	98.50	90.60	98.70	**87.50**
YOLOv11s-seg	35.30	19.50	81.97	98.60	90.70	98.70	**87.50**
**YOLOv12s-seg**	**35.20**	**19.30**	74.63	**98.80**	**90.90**	**99.40**	87.30

Bold values indicate the best performance for each metric. For FLOP and Params, lower values indicate better performance, whereas for FPS and mAP metrics, higher values indicate better performance.

**Figure 4:**
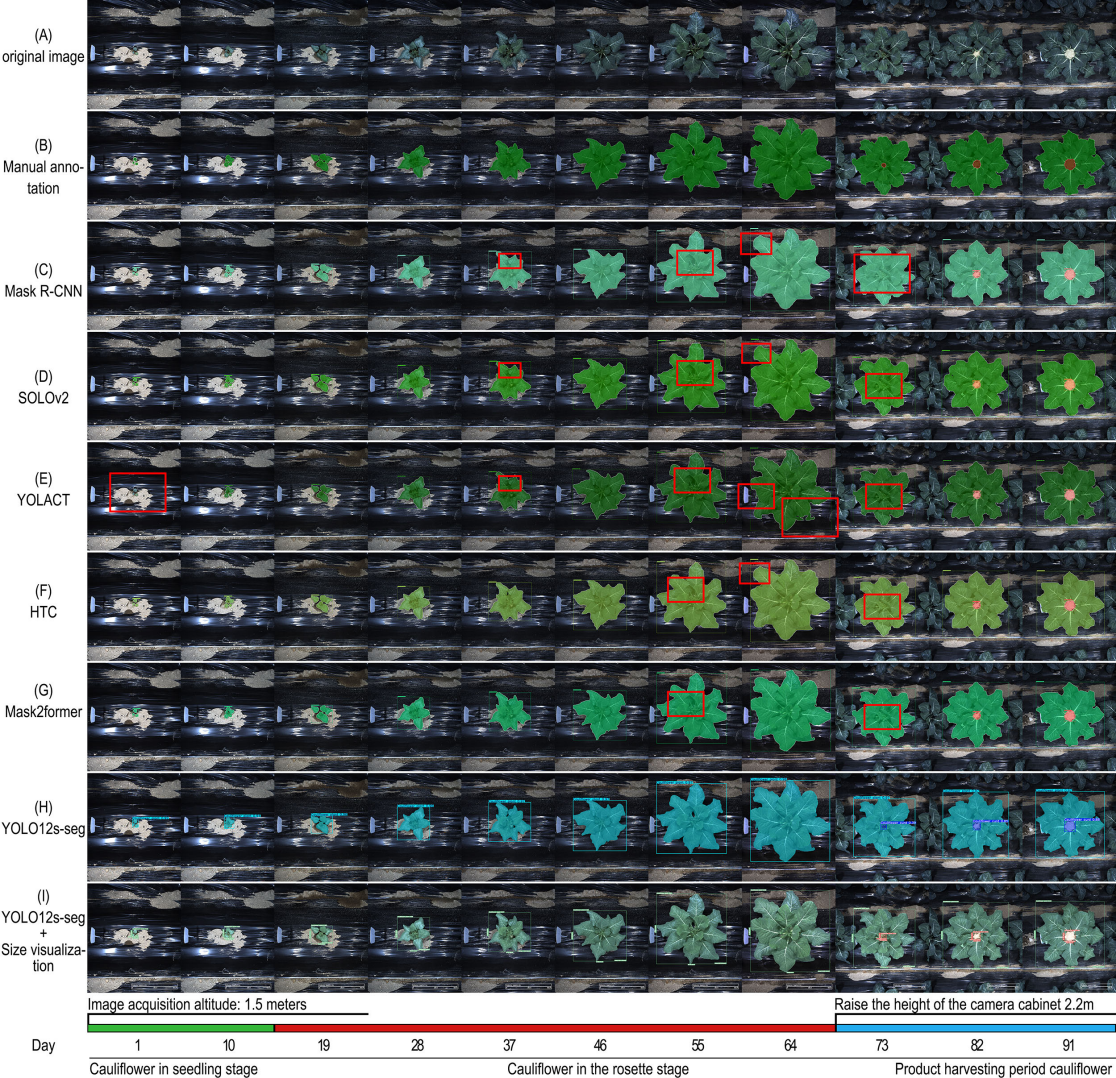
Visual comparison and analysis of segmentation effects of different models: **(A)** Original image; **(B)** Manual annotation image; **(C)** Mask R-CNN instance segmentation image; **(D)** SOLOv2 instance segmentation image; **(E)** YOLACT instance segmentation image; **(F)** HTC instance segmentation image; **(G)** Mask2former instance segmentation image; **(H)** YOLO12s-seg instance segmentation image; **(I)** YOLO12s-seg + morphological index visualization.

However, the performance differences among various models under high-precision metrics deserve attention. As can be seen from the visual prediction results (**Figure 4**) and the quantitative indicators of the YOLO-series models, all models achieved near-perfect segmentation performance for the “cauliflower plant” category but showed obvious limitations on the “cauliflower curd” category. Although the models could reliably locate curds, as reflected by the extremely high mAPbox_50_ values, they still faced challenges in accurately segmenting boundaries occluded by inner leaves. This was indicated by the fact that the mAPmask_50–95_ values of all models were significantly lower than their corresponding mAPmask_50_ values, with a general gap of more than 10 percentage points (for example, the YOLO series dropped from approximately 99% to 87.30%-87.50%). This gap demonstrates that precisely segmenting occluded curd contours is the main difficulty in the current task.

It should be noted that this conclusion mainly applies to partially occluded curds rather than completely invisible targets. Under RGB imaging, fully occluded curds do not provide visible information and therefore cannot be segmented directly. In the present study, the advantage of YOLO12s-seg was reflected in its improved detection of early curds that remained partially visible beneath inner leaves, whereas accurate boundary recovery under severe occlusion remained challenging.

In addition, by comparing the prediction results of continuously growing samples in the same cultivation row (**Figure 4**), it was found that: YOLACT exhibited partial missed detections in small-target recognition at the seedling stage (**Figure 4E**); in terms of segmentation edge precision, Mask R-CNN, SOLOv2, and HTC produced overly smooth edges (**Figures 4C, D**), whereas YOLACT generated overly sharp or even fragmented edges (**Figure 4E**); in the segmentation of internal plant structures, most models treated the plant as a whole, whereas YOLO12s-seg could more accurately identify internal hollow regions (37 d and 56 d). Particularly crucially, for curds in the early curd stage with severe inner-leaf occlusion, YOLO12s-seg showed a unique advantage and successfully detected 73-day-old curds that were missed by all other models.

Based on comprehensive quantitative indicators and visual analysis, YOLOv8s-seg or YOLO12s-seg are the preferred choices when overall performance and deployment efficiency are the primary considerations. For future research aiming to solve the problem of curd segmentation under occlusion, it is necessary to enhance occlusion-specific data or adopt architectures more capable of modeling long-range context, so as to improve the detailed modeling ability for complex boundaries. Overall, YOLO12s-seg exhibits the best comprehensive performance in both visual evaluation and quantitative metrics.

#### 3.1.2 Regression analysis of segmentation accuracy

**Figure 5:**
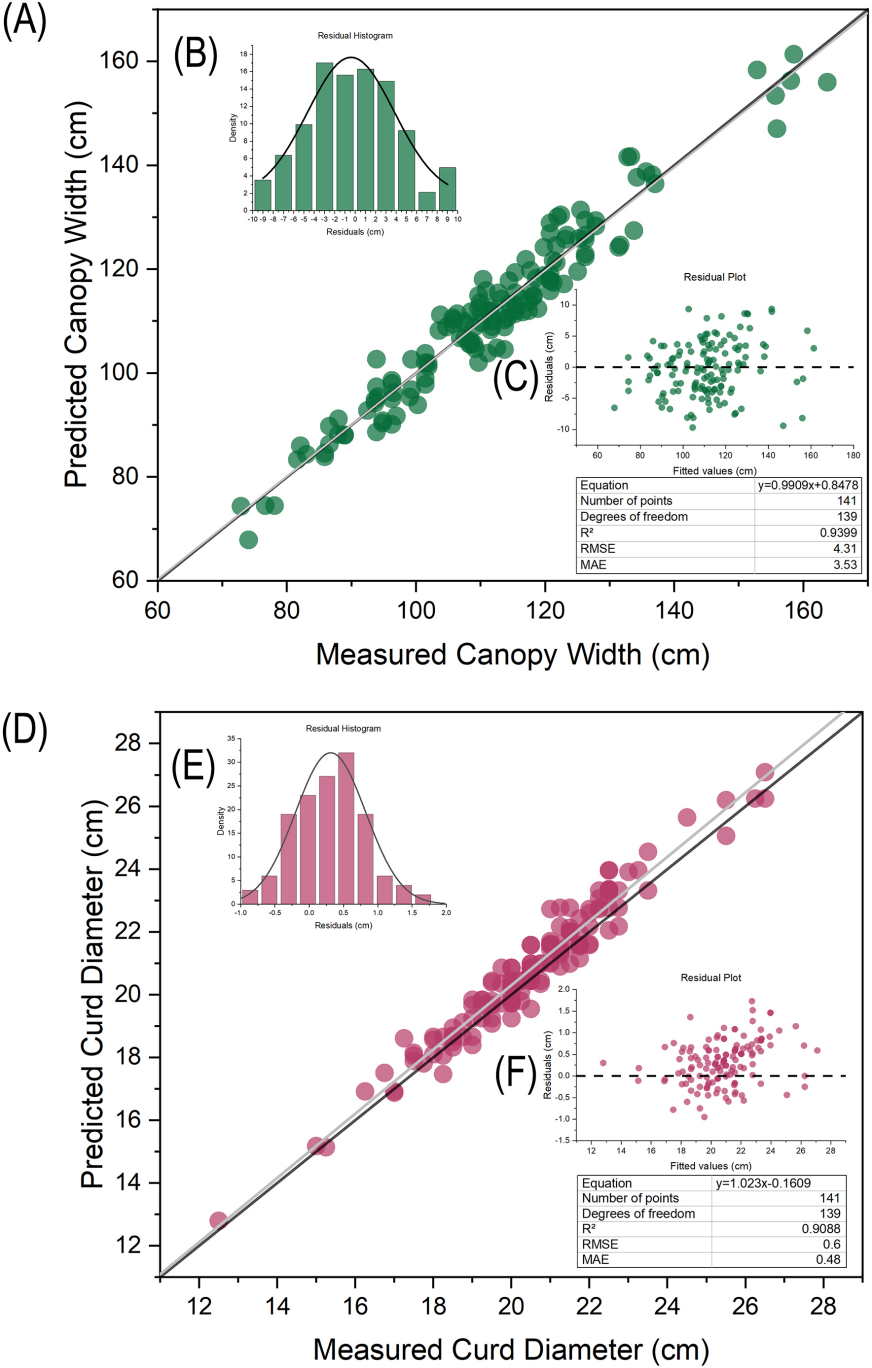
Regression analysis of predicted plant canopy width and curd diameter based on YOLO12s-seg: **(A)** Regression plot of plant canopy width; **(B)** Histogram of plant canopy width prediction residuals; **(C)** Plant canopy width prediction residual plot; **(D)** Regression plot of curd diameter; **(E)** Histogram of curd diameter prediction residuals; **(F)** Curd diameter prediction residual plot.

After evaluating the segmentation accuracy based on the annotated images, to further verify the reliability of image-based phenotypic extraction, this study performed linear regression analysis between the predicted values of plant canopy width and curd diameter obtained by proportional conversion according to camera parameters (object distance, focal length) and the manually measured values (**Figure 5**). The results showed that the scatter points of the predicted values for both plant canopy width and curd diameter were closely distributed on both sides of the fitted curve, with a small deviation between the fitted curve and the 1:1 line. The regression model for plant canopy width (**Figure 5A**) achieved an R^2^ of 0.9399, RMSE of 4.31, and MAE of 3.53; for curd diameter (**Figure 5D**), the R^2^ was 0.9088, RMSE was 0.60, and MAE was 0.48. These results indicate that the phenotypic data of plant canopy width and curd diameter extracted via automatic image segmentation possess high measurement accuracy, can reliably reflect the actual growth status of plants, and are applicable for subsequent growth and development analysis as well as model construction.

### 3.2 Dynamic monitoring and model fitting of cauliflower growth based on the optimal model

#### 3.2.1 Screening of leaf area growth models and evaluation of goodness of fit

**Figure 6:**
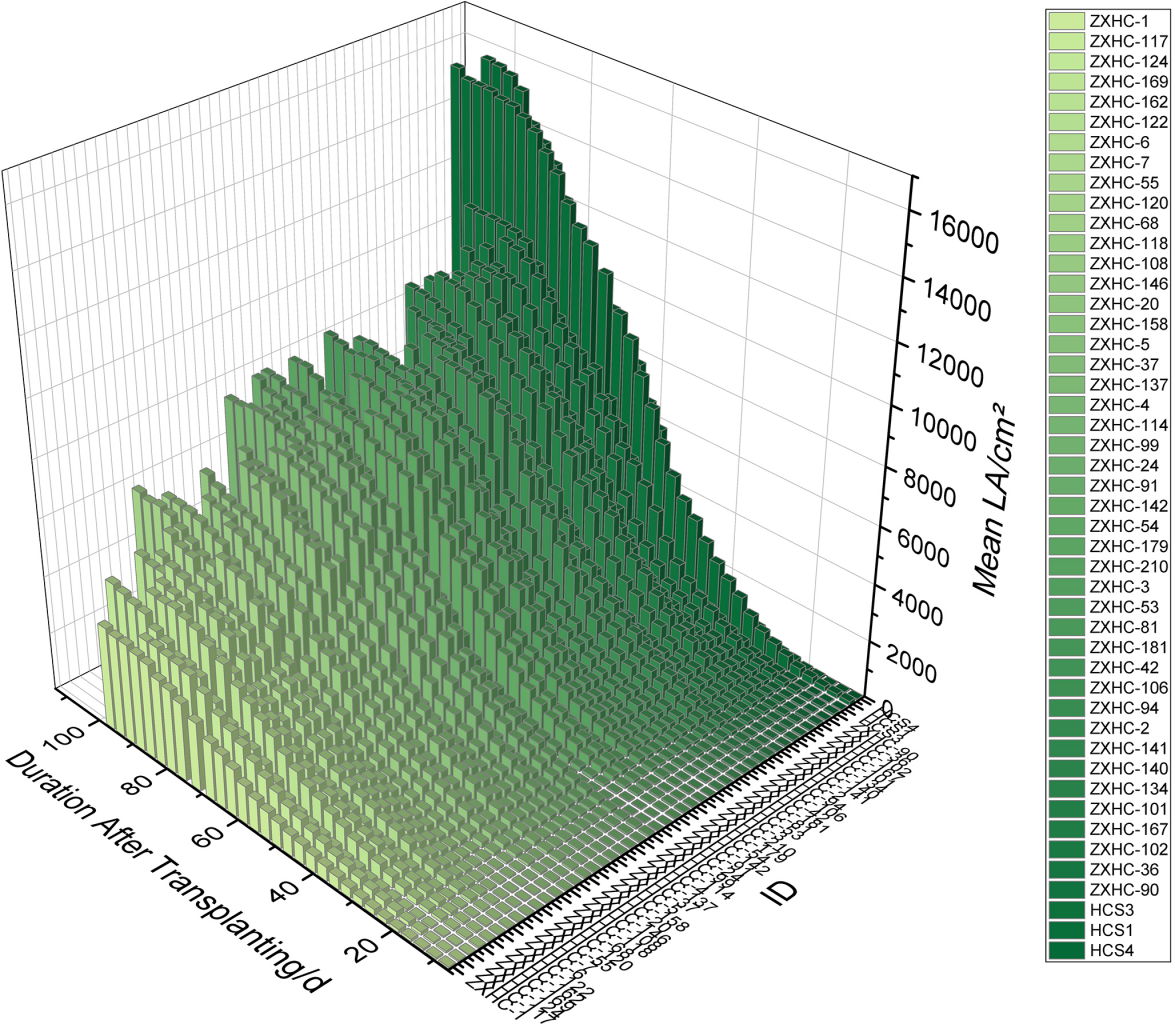
3D histogram of cauliflower population growth phenotypes based on YOLO12s-seg. This image shows the dynamic changes in leaf area during the whole growth period for 47 cauliflower germplasms (N = 141). Data were extracted using YOLO12s-seg and processed by the temporal cut-off method based on sample size: mean values were only calculated within time spans with an effective sample size of N≥2 to ensure population representativeness and eliminate survivor bias caused by abnormally growing individual plants.

Using the optimal model YOLO12s-seg, leaf area growth data were extracted for 47 cauliflower germplasms (141 plants in total) (**Figure 6**). The results showed that the leaf area of different germplasms basically exhibited an S-shaped growth pattern. Therefore, in addition to linear, power, and quadratic functions, three classic S-shaped growth functions—Logistic, Gompertz, and Richards (Archontoulis and Miguez, 2015)—were selected to fit the leaf area growth curves.

The model evaluation results (**Table 4**) showed that the sigmoid growth functions characterizing growth rate were still significantly superior to other models and could more accurately capture the typical “slow-fast-slow” growth dynamics of cauliflower leaf area.

**Table 4:** Goodness-of-fit evaluation of different growth models for cauliflower leaf area expansion curves.

Model name	Model formula	Model evaluation results		
		R^2^	RMSE	Reduced chi-sqr
Linear	LA=at+b	0.9476	945.67	994833.42
Power	$\text{LA}={\text{at}}^{\text{b}}$	0.9737	676.46	554630.68
Quadratic	$\text{LA}={\text{at}}^{2}+\text{bt}+\text{c}$	0.9762	648.56	507571.39
Logistic	$\text{LA}=\frac{\text{a}}{1+{\text{be}}^{-\text{kt}}}$	0.9973	217.83	56384.38
Gompertz	$\text{LA}=\text{a}\left[1+(\text{d}-1){\text{e}}^{-\text{k}\left(\text{x}-\text{xc}\right)}\right]0^{1/\left(1-\text{d}\right)},\text{d}\neq 0$	0.9963	248.53	72153.42
Richards	LA=asin(π(t−b)/c)+d	0.9980	189.21	43879.40

Specifically, the coefficient of determination R^2^ of all three sigmoid functions was higher than 0.996. Among them, the Richards model achieved the highest goodness of fit (R^2^ = 0.9980), with the lowest standard deviation (RMSE = 189.21) and chi-square statistic (Reduced Chi-sqr=43879.40) among all models, indicating excellent consistency and fitting stability between predicted and observed values.

The Logistic model (R^2^ = 0.9973, RMSE = 217.83) and Gompertz model (R^2^ = 0.9963, RMSE = 248.53) also exhibited excellent fitting performance. In contrast, the linear, power function, and quadratic function models had significantly higher RMSE and Reduced Chi-sqr values, especially the Chi-square value, which was nearly one order of magnitude higher than that of the sigmoid functions. This suggests that although these models can roughly describe the increasing trend, they fail to accurately reflect the saturation characteristic during the sigmoid growth process and the underlying biological mechanism.

In summary, the Richards model performed best in terms of R^2^, RMSE, and Reduced Chi-sqr, providing the most reliable mathematical framework for describing the temporal growth pattern of cauliflower leaf area across the whole growth period.

#### 3.2.2 Extraction of key plant growth stages based on the richards model

**Figure 7:**
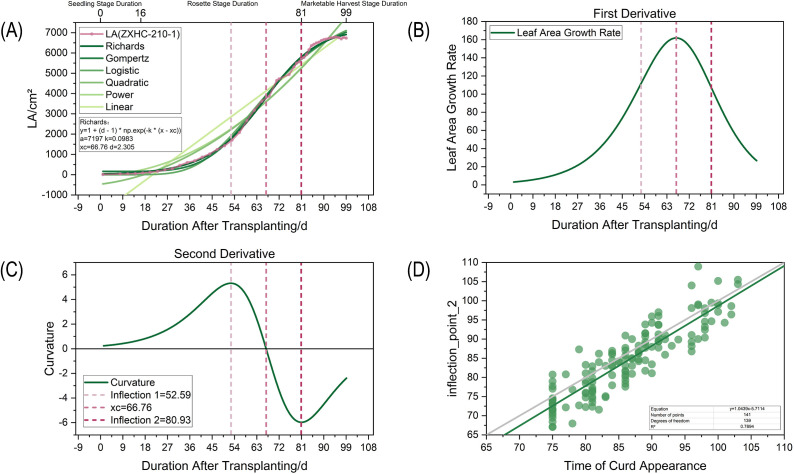
Growth kinetic analysis based on leaf area growth model fitting: **(A)** Fitting comparison of six growth models to leaf area data (using germplasm ZXHC-210 as an example); **(B)** First-order derivative curve based on the optimal Richards model (reflecting growth rate); **(C)** Second-order derivative curve and identification of two inflection points used to define the rapid growth period; **(D)** Linear regression analysis between Inflection point 2 and manually observed curd appearance time.

Based on the optimal Richards model selected, we further quantified the rapid growth period of leaf area by analyzing the derivative characteristics of the fitted curve (Shabani et al., 2018). Specifically, the first- and second-order derivatives of the fitted curve were calculated, where two inflection points (inflection point 1 and inflection point 2) at which the second-order derivative equals zero defined the interval during which the growth rate accelerated continuously. The width of this interval was defined as the “duration of the rapid leaf area growth period” (Xu et al., 2018). Taking plant ZXHC-210–1 as an example (**Figure 7A**), the Richards model fitted the actual growth data well and accurately captured the sigmoidal growth trend of “slow-fast-slow”. The first-order derivative curve in (**Figure 7B**) showed a parabolic shape, with its vertex corresponding to the maximum leaf area growth rate. The second-order derivative curve in (**Figure 7C**) clearly identified two inflection points, and the interval between them represented the rapid growth period. The model parameters and analysis results for other plants are presented in **Table A1**.

Notably, regression analysis (**Figure 7D**) showed that Inflection point 2 (the end point of the rapid leaf area growth period) had a significant linear relationship with the manually observed curd appearance time (y = 1.0439x − 5.7114, R^2^ = 0.7894). The negative intercept of this model indicated that Inflection point 2 systematically preceded the externally observed curd appearance time. Combined with the biological fact that curds are often occluded by inner leaves during early development, we speculate that this turning point in leaf area growth may represent an earlier physiological initiation moment when curds begin rapid expansion, rather than the time of visible morphological appearance. Therefore, Inflection point 2 provides a potential and earlier indicator for predicting the key transition of curd development. The specific average time difference between them (approximately 5.71 days) needs further verification with more samples and more precise physiological observations.

#### 3.2.3 Screening and goodness-of-fit evaluation of curd area growth models

**Figure 8:**
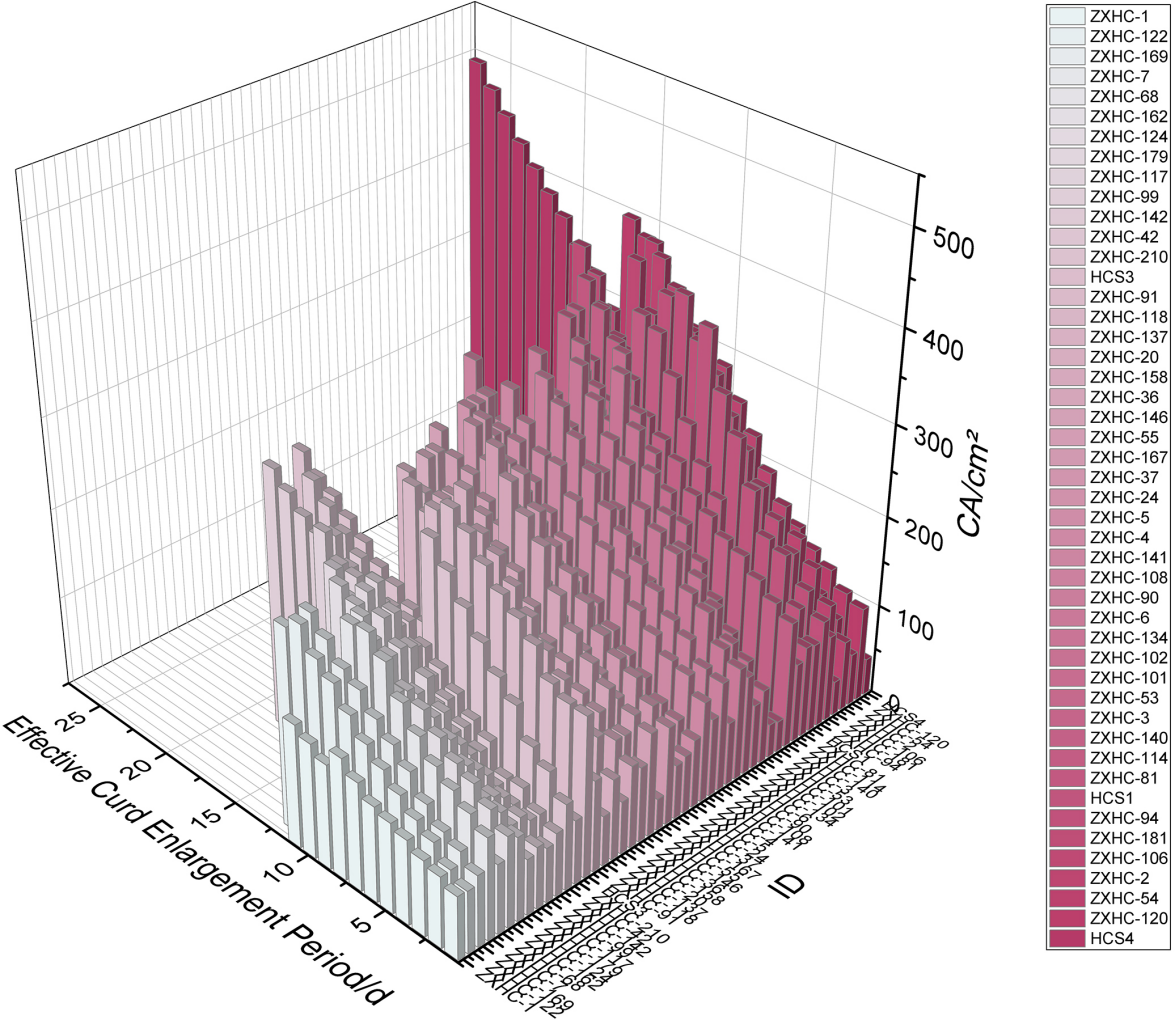
Growth dynamics of curds in 47 cauliflower germplasms based on development alignment. Values represent the truncated means of three biological replicates. All growth curves were uniformly aligned to the curd appearance time (Day 1) to eliminate differences in maturity among germplasms and synchronize the developmental process.

Using the same optimal model YOLO12s-seg, images of 47 germplasms at the commercial harvest stage were processed to extract curd area data across the full time series. As shown in **Figure 8**, the curd area growth pattern differed from that of leaf area: it did not show an S-shaped growth trend from curd appearance to the optimal harvest stage. Therefore, only four models were selected for comparison: linear, power function, quadratic function, and exponential function (**Table 5**).

**Table 5:** Comparison of fitting performance of different mathematical models for cauliflower curd area growth dynamics.

Model name	Model formula	Model evaluation results		
		R^2^	RMSE	Reduced Chi-sqr
Linear	CA=at+b	0.9784	16.87	347.71
Power	$\text{CA}={\text{at}}^{\text{b}}$	0.9585	22.67	562.10
Quadratic	$\text{CA}={\text{at}}^{2}+\text{bt}+\text{c}$	0.9920	10.10	121.56
Sine	$\text{CA}={\text{y}}_{0}+\text{A}\sin\left(\text{π}\frac{\left(\text{t}-\text{tc}\right)}{\text{w}}\right)$	0.9929	10.10	120.99
Exponential	$\text{CA}={\text{ae}}^{\text{bt}}$	0.9239	17.87	1642.69

Based on the fitting analysis of curd growth curves for a total of 141 plants from 47 cauliflower germplasms, significant differences were observed in the goodness of fit among the models. The evaluation results (**Table 5**) showed that the Sine model achieved the highest coefficient of determination (R^2^ = 0.9929), with the lowest standard deviation (RMSE = 10.10) and chi-square value (Reduced Chi-sqr = 120.99) among all models, indicating that this model provided the most accurate and stable description of the curd area growth process over time. The quadratic polynomial model (R^2^ = 0.9920, RMSE = 10.10, Reduced Chi-sqr = 121.56) exhibited a similarly high fitting performance. The linear model (R^2^ = 0.9784) and power function model (R^2^ = 0.9585) showed relatively lower goodness of fit, with considerably higher RMSE and Reduced Chi-sqr values than the former two models. The exponential model performed the weakest (R^2^ = 0.92), and its extremely high Reduced Chi-sqr value (1642.69) further confirmed its limited ability to interpret such growth dynamics.

In comprehensive comparison, the Sine model performed best in all three evaluation indices (R^2^, RMSE, and Reduced Chi-sqr), which further verified its reliability as the optimal model for fitting cauliflower curd growth dynamics, providing a mathematical foundation for subsequent growth stage quantification and phenotypic analysis based on this model.

#### 3.2.4 Growth classification analysis of cauliflower heads based on sine functions

Constructing growth kinetic models is an effective approach to quantifying the developmental processes of horticultural crops and predicting key phenological stages. In simulating the development and harvest stages of various horticultural crops, using the sine function to describe temperature response patterns has been proven superior to linear models, and multi-model integration can effectively optimize prediction pathways (Cheng et al., 2023). Meanwhile, studies on the growth heterogeneity among different varieties are often conducted by fitting unified growth curves (e.g., the Logistic curve) and comparing their key parameters such as heat requirement (Cifuentes-Carvajal et al., 2024).Furthermore, building statistical models using crop phenological records and meteorological data has become an important method for predicting growth duration and yield (Mortlock et al., 2025). These studies provide a methodological foundation for variety classification based on full-time-series growth data. However, most of them focus on time-driven prediction, and research on the fine classification of harvest potential directly based on the morphological characteristics of growth curves themselves (e.g., trends of rate changes) remains insufficient.

To investigate the heterogeneity of curd growth curves among different cauliflower germplasms, this study adopted the sine function model ( $\text{CA}={\text{y}}_{0}+\text{A}\sin\left(\text{π}\left(\text{t}-\text{tc}\right)/\text{w}\right)$) to fit and analyze 141 full-time-series growth curves of cauliflower curd area, and established a multi-criteria classification method based on growth kinetic characteristics. This method classified curd growth status into three types: Mature and Full type (growth rate decreased significantly, at the post-peak stage), Peak Burst type (growth rate reached the peak and remained stable, at the peak plateau stage), and Steady Climbing type (growth rate continued to rise, at the pre-peak stage). The classification algorithm comprehensively considered parameters such as the growth rate trend and the ratio of harvest-time growth rate to maximum growth rate. It aims to accurately quantify the growth vigor of curds at harvest.

**Figure 9:**
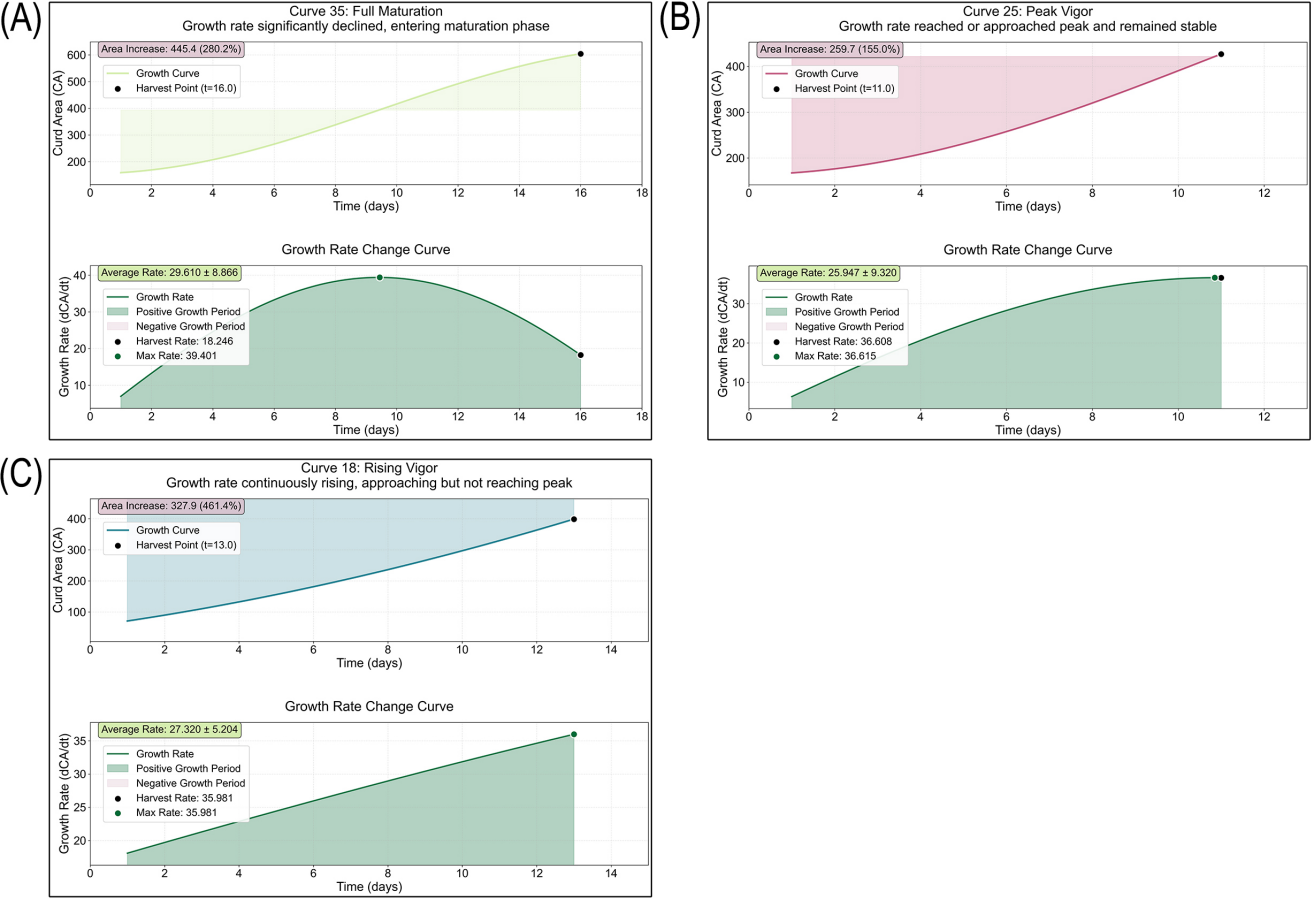
Typical curves of cauliflower curd growth kinetic classification based on sine function fitting: **(A)** Mature & Full type; **(B)** Peak Burst type; **(C)** Steady Climbing type.

The classification results (**Figure 9**, **Table A2**) showed that curd growth status exhibited significant heterogeneity: the Steady Climbing type accounted for the highest proportion (68.8%, 97/141), with a continuously increasing growth rate and still in the rapid growth stage at harvest (**Figure 9C**). The harvest-time growth rate ranged from 14.45 to 79.20, and the growth duration ranged from 5.0 to 29.0 days; the Mature and Full type ranked second (29.1%, 41/141), characterized by a significantly decreased growth rate at harvest and entering the physiological maturity stage (**Figure 9A**). The harvest-time growth rate ranged from 1.98 to 49.58, and the growth duration ranged from 6.0 to 26.0 days; the Peak Burst type accounted for the lowest proportion (2.1%, 3/141), characterized by curds exactly at the peak plateau of growth rate at harvest (**Figure 9B**). The harvest-time growth rate ranged from 27.90 to 36.61, and the growth duration ranged from 7.0 to 11.0 days.

The core value of this classification method is to provide a quantitative evaluation tool for determining the harvest time of cauliflower curds. By identifying the growth stage of curds, producers can more accurately grasp the harvest window: for Steady Climbing curds, appropriate delayed harvest can achieve higher yield; for Peak Burst curds, the current time is the optimal commercial harvest stage; for Mature and Full curds, timely harvest should be conducted to avoid quality deterioration. This method can be directly applied in production practice to assist in judging the growth vigor of curds at harvest and optimize harvest decisions.

### 3.3 Construction of cauliflower trait extraction and analysis platform based on the optimal model

**Figure 10:**
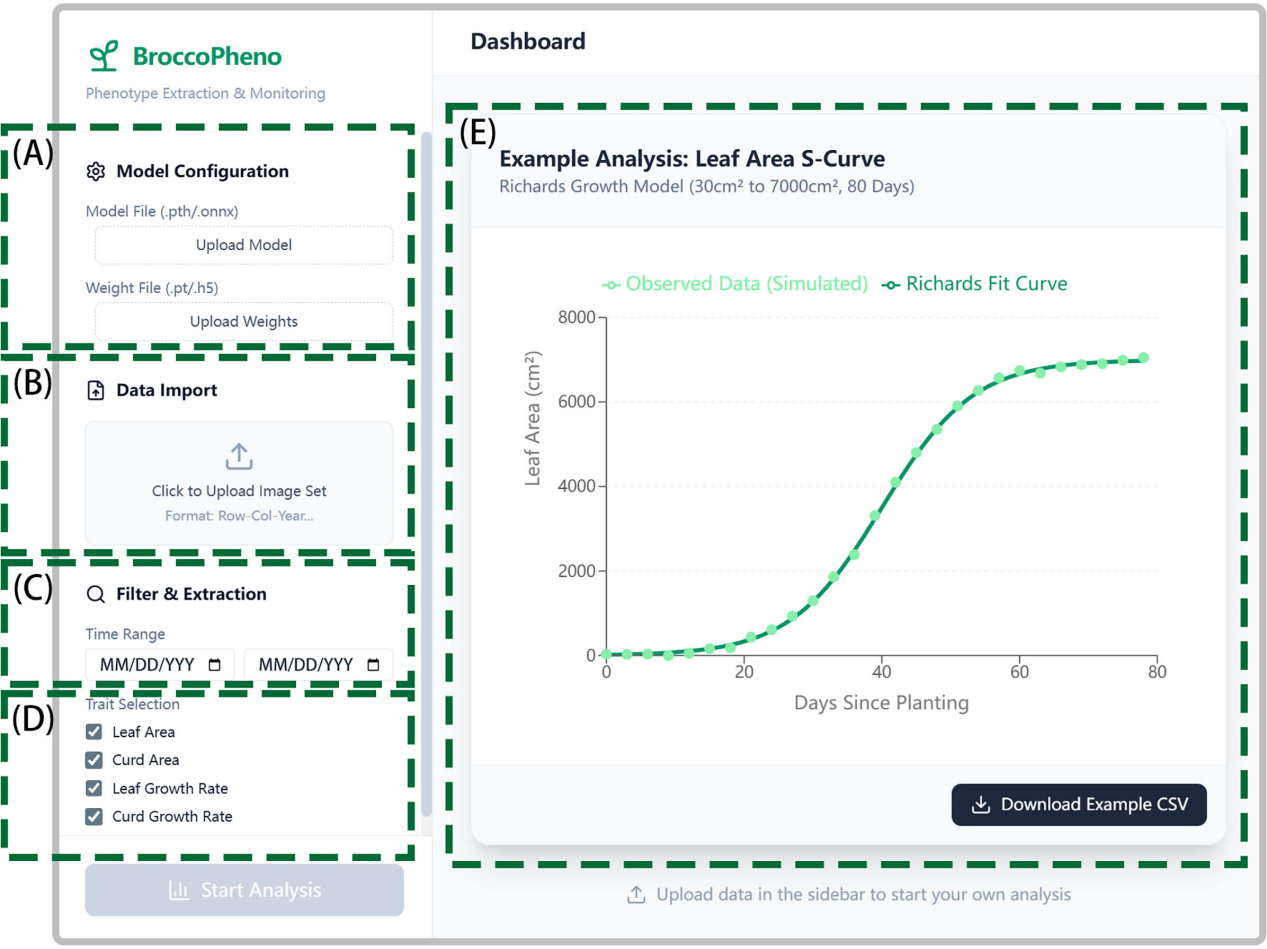
Intelligent monitoring and phenotypic analysis platform for cauliflower growth dynamics: **(A)** Model configuration module; **(B)** Data import module; **(C)** Time filtering module; **(D)** Phenotypic trait extraction and definition; **(E)** Visualization dashboard and decision output.

Based on YOLO12s-seg, the optimal model for cauliflower plant and curd segmentation screened in the previous stage, this study developed an image analysis application for growth monitoring (**Figure 10**). The application allows users to select the model and upload the trained weight files (**Figure 10A**), which facilitates model updating and endows it with the potential to be extended to other crops. Users are required to name and upload images following the format of “row_column_year_month_day_acquisition_times” (**Figure 10C**), and select the image acquisition height and analysis time interval (**Figure 10B**). The application can automatically extract key traits within the specified period, including leaf area, leaf area growth rate, curd area, and curd area growth rate (**Figure 10D**). Its “Leaf Area Analysis” function fits time-series leaf area data using the Richards function and automatically identifies the rapid growth interval of leaf area via derivative analysis; the “Curd Analysis” function fits the curd growth curve using the sine function, and then intelligently classifies curd status into Steady Climbing type, Peak Burst type, or Mature & Full type according to growth kinetic characteristics, providing a quantitative basis for judging harvest time. All analysis results can be finally summarized and exported into a structured CSV file (**Figure 10E**) for easy download and subsequent utilization. This application integrates DL segmentation, growth curve modeling, and agronomic analysis, providing an intuitive and operable tool for quantitative monitoring and decision support of the cauliflower growth process.

### 3.4 Analysis and clustering of growth phenotypic differences among germplasms

Based on the high-throughput phenotypic traits extracted above, we further explored the coordination between vegetative growth (leaves) and reproductive growth (curds) in cauliflower, and revealed the intrinsic classification of growth types through unsupervised clustering.

#### 3.4.1 Correlation analysis

**Figure 11:**
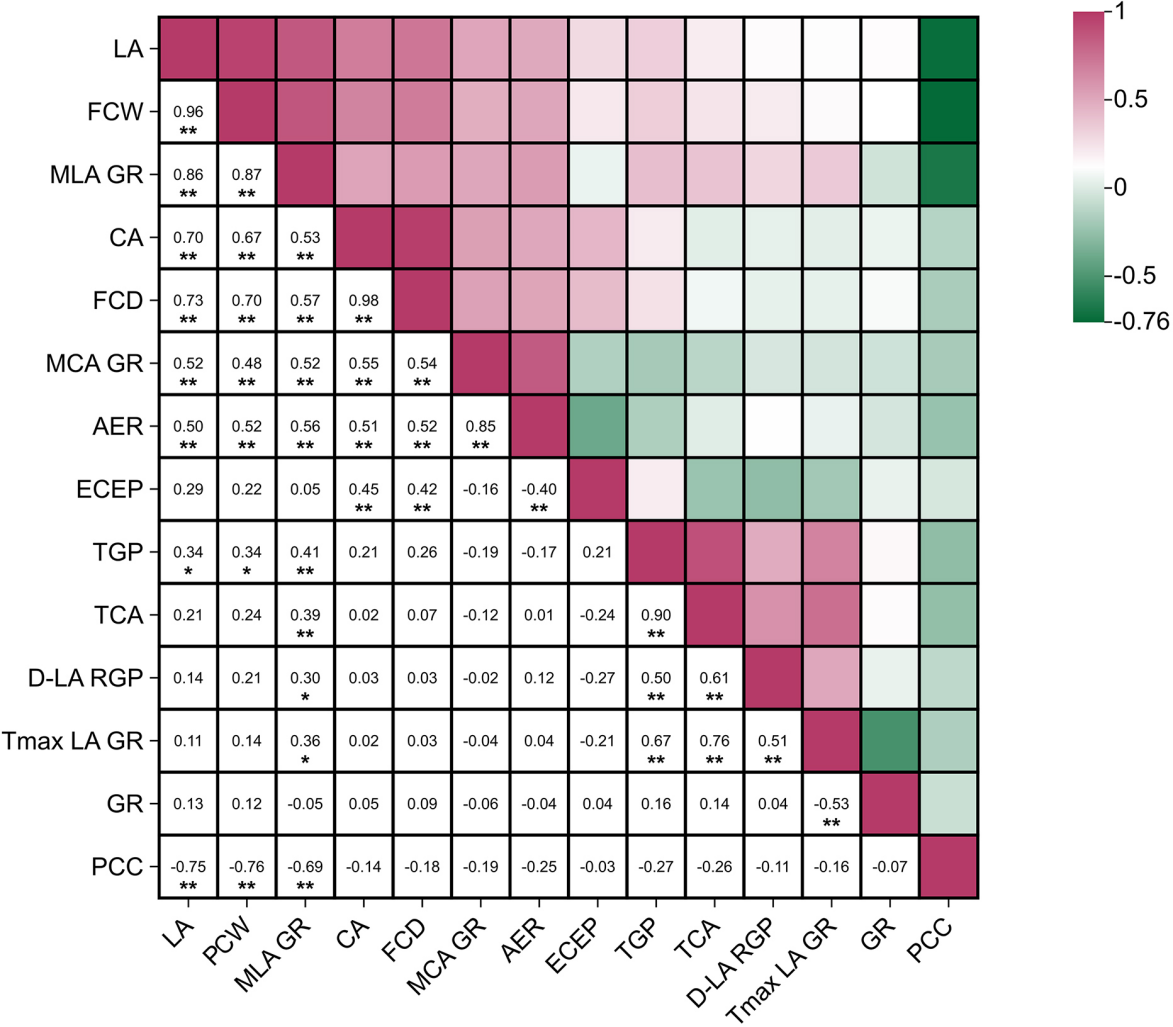
Correlation analysis between phenotypic traits and growth kinetic parameters of cauliflower plants. * indicates significant correlation at the 0.05 level, ** indicates significant correlation at the 0.01 level. LA, Leaf area; PCW, plant canopy width; MLA GR, maximum leaf area growth rate; CA, curd area; FC, curd diameter; MCA GR, maximum curd area growth rate; AER, average curd expansion rate; ECEP, effective curd expansion period; TGP, total growth period; TCA, curd appearance time; D-LA RGP, duration of rapid growth period of leaf area; Tmax LA GR, time to reach maximum leaf area growth rate; GR, growth rhythm; PCC, plant-curd coordination.

Correlation analysis of the main growth and yield traits in cauliflower showed extensive and varying degrees of correlation among traits (**Figure 11**). In this analysis, PCW, LA, CA, and FCD refer to the final observed values of these traits for each germplasm. Vegetative growth traits exhibited high synergy: the correlation coefficient between leaf area (LA) and plant canopy width (PCW) reached 0.96, and both were strongly positively correlated with the maximum leaf area growth rate (MLA GR), with r values of 0.86 and 0.87, respectively, indicating that leaf area growth rate was highly consistent with final canopy size.

Curd traits were highly consistent internally, with curd area (CA) almost perfectly positively correlated with curd diameter (FCD) (r = 0.98). Curd size was moderately positively correlated with vegetative growth scale; the correlation coefficients of final curd area with final leaf area and final plant canopy width were 0.70 and 0.67, respectively, suggesting that larger vegetative organs generally favored the formation of larger curds. In terms of growth dynamics, total growth period (TGP) was strongly positively correlated with curd appearance time (TCA) (r = 0.90), and both were closely related to the time to reach maximum leaf area growth rate (Tmax LA GR). Notably, effective curd expansion period (ECEP) was negatively correlated with average curd expansion rate (AER) to a certain extent (r = -0.4), implying that individuals with a faster expansion rate might have a shorter effective expansion period.

A key finding was that plant-curd coordination (PCC, defined as final curd area/final leaf area) was significantly negatively correlated with vegetative growth scale and rate, with correlation coefficients of -0.75, -0.76 and -0.69 with final leaf area, final plant canopy width and maximum leaf area growth rate, respectively. This result indicated a competitive allocation of assimilates between vegetative and reproductive growth in plants, which was consistent with previous studies (Kage et al., 2003; Hu et al., 2025). In addition, some traits such as growth rhythm (GR) and effective curd expansion period showed weak correlations with most other traits, which may stem from the intrinsic regulatory independence of these traits. Previous studies have shown that curd initiation time itself is a key developmental event sensitive to environmental factors (such as temperature) and can be independently regulated (Lin et al., 2019). Meanwhile, recent genomic studies have revealed that curd formation in cauliflower involves a relatively specialized gene regulatory network (Chen et al., 2024), which may make its expansion process independent of the genetic pathways governing vegetative growth. Therefore, it is reasonable that GR, as a composite trait integrating environmentally regulated reproductive initiation and vegetative growth dynamics, shows relative independence; and as a core stage of reproductive growth, the specificity of the genetic regulatory pathway of effective curd expansion period also supports the observation of its weak correlation with most other traits.

In summary, there is a complex coordination and trade-off relationship between vegetative and reproductive growth in cauliflower. In high-yield breeding or cultivation, on the basis of ensuring appropriate vegetative growth, emphasis should be placed on optimizing the allocation efficiency of assimilates and improving plant-curd coordination to achieve superior economic yield.

#### 3.4.2 Germplasm clustering analysis based on growth curve characteristics

**Figure 12:**
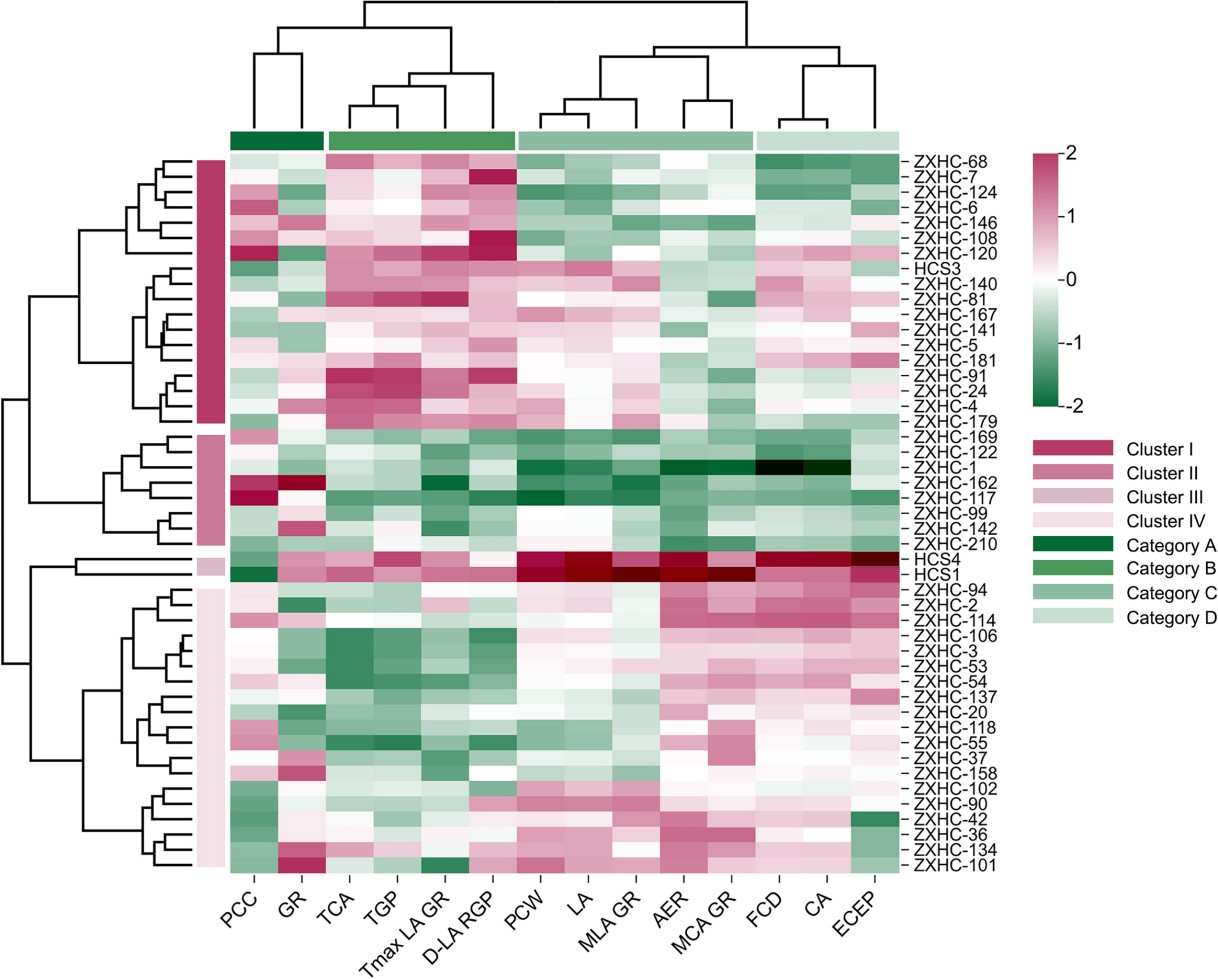
Heatmap of hierarchical clustering analysis of growth traits and kinetic parameters of 47 cauliflower germplasms. PCC, plant-curd coordination (final curd area/final leaf area); GR, growth rhythm (curd appearance time/time to reach maximum leaf area growth rate); TCA, curd appearance time; TGP, total growth period; Tmax LA GR, time to reach maximum leaf area growth rate; D-LA RGP, duration of rapid growth period of leaf area; PCW, plant canopy width; LA, Leaf area; MLA GR, maximum leaf area growth rate; AER, average curd expansion rate; MCA GR, maximum curd area growth rate; FCD, curd diameter; CA, curd area; ECEP, effective curd expansion period.

Cluster analysis of 47 cauliflower germplasms (**Figure 12**) showed that all germplasms could be divided into 4 distinct groups based on growth and yield traits. Meanwhile, the 14 evaluated traits themselves were clustered into 4 trait groups with clear biological significance, jointly revealing the structured diversity of the germplasm resources. For the size-related traits, the clustering analysis was based on the final observed values of PCW, LA, CA, and FCD.

At the germplasm level, the four groups exhibited clear differentiation. Group I (Comprehensive Coordinated Type) had most traits at medium levels, with the most balanced growth rhythm (curd appearance time/time to reach maximum leaf area growth rate = 1.10) and relatively high plant-curd coordination (4.72%), indicating coordinated transition between vegetative and reproductive growth and reasonable biomass allocation. Group II (Mid-maturity Compact Type) had the smallest overall size among the four groups, with the shortest growth period (95.00 days). Final leaf area (5581.94 cm^2^), plant canopy width (95.13 cm), and curd area (263.56 cm^2^) were significantly lower than those of other groups, characterized by early curd appearance (81.79 days) and compact plant architecture. Group III (Large High-Yield Type) showed absolute superiority in all growth and yield indicators, with the highest final leaf area (15423.59 cm^2^), maximum leaf area growth rate (706.66 cm^2^/day), final curd area (467.26 cm^2^), and maximum curd area growth rate (62.01 cm^2^/day). However, its plant-curd coordination was the lowest (3.04%), reflecting that vigorous vegetative growth was not efficiently converted into a relatively high proportion of curd. Group IV (Curd-Dominant Type) had moderate vegetative growth scale but outstanding curd expansion dynamics. The maximum curd area growth rate (48.23 cm^2^/day) and average curd expansion rate (25.30 cm^2^/day) were second only to Group III, while the effective curd expansion period was the shortest (11.51 days), showing a “rapid formation” pattern, which resulted in a significantly larger final curd area (354.57 cm^2^) than Groups I and II.

At the trait clustering level, the 14 indicators were grouped into four functional clusters: (1) Source-sink Coordination Traits, including plant-curd coordination and growth rhythm, reflecting reproductive transition efficiency; (2) Growth Period Traits, including curd appearance time, total growth period and related durations, determining the phenological process; (3) Growth Vigor Traits, covering final organ size and growth rates of leaves and curds, representing growth intensity; (4) Curd Formation Traits, including curd area, curd diameter and effective curd expansion period, which mainly characterize curd morphogenesis. This classification has inherent biological logic: first, the independence of the “Curd Formation Traits” is directly supported by recent studies, confirming that curd morphology is an independent phenotypic regulatory module (Xiao et al., 2024), governed by a relatively specialized genetic network (Chen et al., 2024). Second, the organ size and yield indicators included in the “Growth Vigor Traits” often show coordinated changes in cultivation experiments (Feng et al., 2023). Finally, the division of the “Source-sink Coordination Traits” is supported by advanced theories of plant physiology, in which the material allocation and coordination between source and sink constitute a core independent process affecting yield (Rosado-Souza et al., 2023).

**Figure 13:**
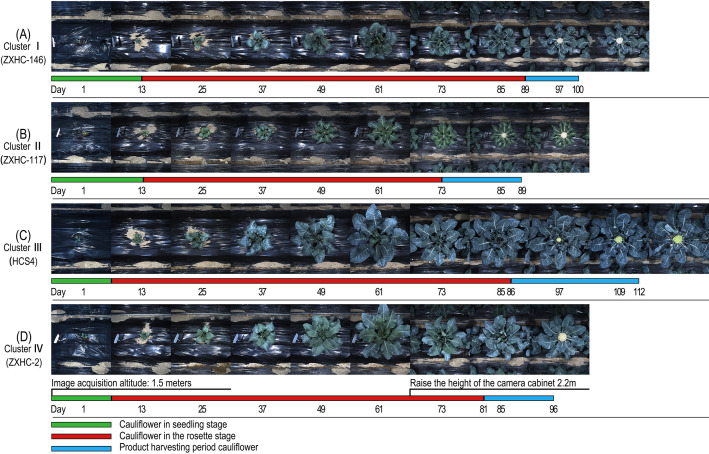
Comparison of growth processes of four typical cauliflower varieties based on cluster analysis: **(A)** Cluster I (Comprehensively coordinated type), ZXHC-146; **(B)** Cluster II (Mid-maturity compact type), ZXHC-117; **(C)** Cluster III (Large high-yield type): HCS4; **(D)** Cluster IV (Curd-dominant type), ZXHC-2.

The two clustering results corroborated each other, indicating that the differences among germplasm types were essentially the result of the coordinated expression of different trait groups. To visually demonstrate the growth characteristics of each group, this study selected representative germplasms from each group — ZXHC-146 (Group I), ZXHC-117 (Group II), HCS4 (Group III), and ZXHC-2 (Group IV) — and analyzed them by comparing images of their entire growth processes side by side (**Figure 13**). The images showed that ZXHC-146 had relatively balanced durations across growth stages, with proportional seedling, rosette, and commercial harvest stages, consistent with its “Comprehensive Coordinated Type” characteristic. ZXHC-117 had a short overall growth period, a rapid transition from the rosette stage to curd appearance, and a small vegetative growth scale, matching its description as “Mid-maturity Compact Type”. HCS4 exhibited a significantly prolonged vegetative growth stage, vigorous leaf growth, and rapid curd expansion but a low proportion of curd expansion relative to the total growth period, verifying its feature of “large and high-yielding but low plant-curd coordination”. ZXHC-2 showed short seedling and rosette stages, with an early and concentrated curd expansion period, highlighting its group attribute of “curd-dominant and rapid formation”. Colored line segments below the images marked the duration of each stage, clearly reflecting the significant differences among the four germplasm groups in phenological progression and growth allocation, which were highly consistent with the trait grouping and germplasm classification obtained from the cluster analysis.

Detailed trait data of the 47 germplasms, mean trait values of each group, and phenotypic comparison plots of all germplasms at commercial harvest stage are presented in **Table A3** and **Figure A1, A2**.

## 4 Discussion

This study established a complete high-throughput phenotyping analysis framework for cauliflower, from precise image segmentation to growth dynamic analysis. The core findings and contributions are reflected in three aspects: methodology, agronomic understanding, and application tools.

Methodologically, this study verified the feasibility of achieving high-throughput trait extraction throughout the entire growth period of cauliflower using a lightweight instance segmentation model. The selected YOLO12s-seg model achieved nearly perfect segmentation accuracy for plants under sparse planting conditions (mAPmask_50_ > 99%), which may be attributed to its efficient network architecture and optimized feature fusion mechanism (Khanam and Hussain, 2025), enabling excellent target contour capture while maintaining high-speed inference. However, the decreased segmentation accuracy for curds occluded by inner leaves (significantly reduced mAPmask_50-95_) precisely reveals a common bottleneck of current visual phenotyping techniques in complex field scenarios: limited ability to handle occlusion and blurred boundaries. This points to directions for future research: model optimization should focus more on enhancing contextual understanding and detailed modeling rather than merely pursuing overall accuracy improvement (Badgujar et al., 2024; Kanna et al., 2024).

At the level of agronomic understanding, this study transformed continuous image data into growth parameters with clear biological significance through growth model fitting and kinetic analysis. The research not only validated the excellent fitting performance of the Richards model for the S-shaped growth of leaf area (R^2^ = 0.998) but, more importantly, found via derivative analysis that the inflection point marking the end of the rapid leaf area growth period (Inflection point 2) occurred systematically earlier than the manually observed curd appearance time. This raises a valuable hypothesis: this inflection point may indicate the initiation of the physiological process of curd expansion rather than its visible morphological appearance, which is critical for selecting the transition node from vegetative to reproductive growth in cauliflower (Chen et al., 2024). It also provides a potential and more sensitive quantitative indicator for early prediction of reproductive transition using vegetative growth dynamics. In addition, the classification of curd growth status based on the sine model (Mature & Full type, Peak Burst type, Steady Climbing type) advances traditional harvest-stage judgment from empirical observation to quantitative decision-making based on growth rate, with direct guiding significance for production. More broadly, this also illustrates that dynamic phenotypic descriptors provide information beyond final trait values, because they capture developmental timing, growth rhythm, and harvest potential that are directly relevant to both breeding selection and cultivation management.

At the application level, the integrated analysis platform developed in this study combines segmentation, fitting, classification, and visualization functions, significantly lowering the technical threshold and providing a convenient tool for breeders and producers. Through cluster analysis of full-time-series phenotyping data of 47 germplasms, this study not only validated the traditional growth-period-centered classification (e.g., mid-maturity, late-maturity) used in breeding (Zhu et al., 2022; Xiao et al., 2024) but also expanded the classification dimensions using high-throughput continuous dynamic traits. It revealed significant differentiation in source (vegetative organ)-sink (reproductive organ) coordination among cauliflower germplasms and identified functionally oriented groups such as “Comprehensive Coordinated Type” and “Curd-Dominant Type” that go beyond maturity. This provides finer and more comprehensive phenomics evidence for parental selection and targeted breeding.

Despite the promising results, several limitations of the present study should be acknowledged. First, the current framework was based only on RGB images. Therefore, it cannot recover information from completely occluded curds, and segmentation performance may decline under severe inner-leaf occlusion. Future studies may combine RGB imaging with other sensing modalities, such as hyperspectral or infrared imaging, to improve the detection of early curd development and to enable the acquisition of additional physiological traits. Second, the dataset used in this study was collected in a single greenhouse environment and within one growing season. Thus, the robustness and generalizability of the trained model under different environments, planting densities, and imaging systems remain to be further evaluated.

## 5 Conclusions

This study established a high-throughput phenotyping framework for whole-growth-period monitoring of cauliflower based on instance segmentation and time-series analysis. Among the evaluated models, YOLO12s-seg achieved the best overall balance between segmentation accuracy and inference efficiency, and the image-derived plant canopy width and curd diameter showed high agreement with manual measurements. Based on the extracted time-series traits, the Richards model effectively described leaf area dynamics, while the Sine model provided the best fit for curd area dynamics. Derivative analysis further suggested that the end of the rapid leaf-area growth phase may serve as an earlier indicator of curd developmental transition than visible curd appearance. In addition, curd growth patterns at harvest were quantitatively classified into three kinetic types, and 47 germplasms were grouped into four phenotypic categories according to dynamic growth traits. The developed analysis platform integrates image segmentation, trait extraction, dynamic modeling, and decision support, providing a practical tool for cauliflower phenotyping and harvest evaluation. Overall, this work offers an automated solution for dynamic phenotyping of cauliflower and provides a useful analytical framework for breeding and growth monitoring in other crops.

## Data Availability

The raw data supporting the conclusions of this article will be made available by the authors, without undue reservation.
