# Supplementary figures and images for: High-throughput phenotypic analysis of plant and curd growth dynamics during the whole growth period of cauliflower based on instance segmentation
Source: Front Plant Sci. 2026 May 13;17:1836813. doi: 10.3389/fpls.2026.1836813 (PMC13212518; doi:10.3389/fpls.2026.1836813)

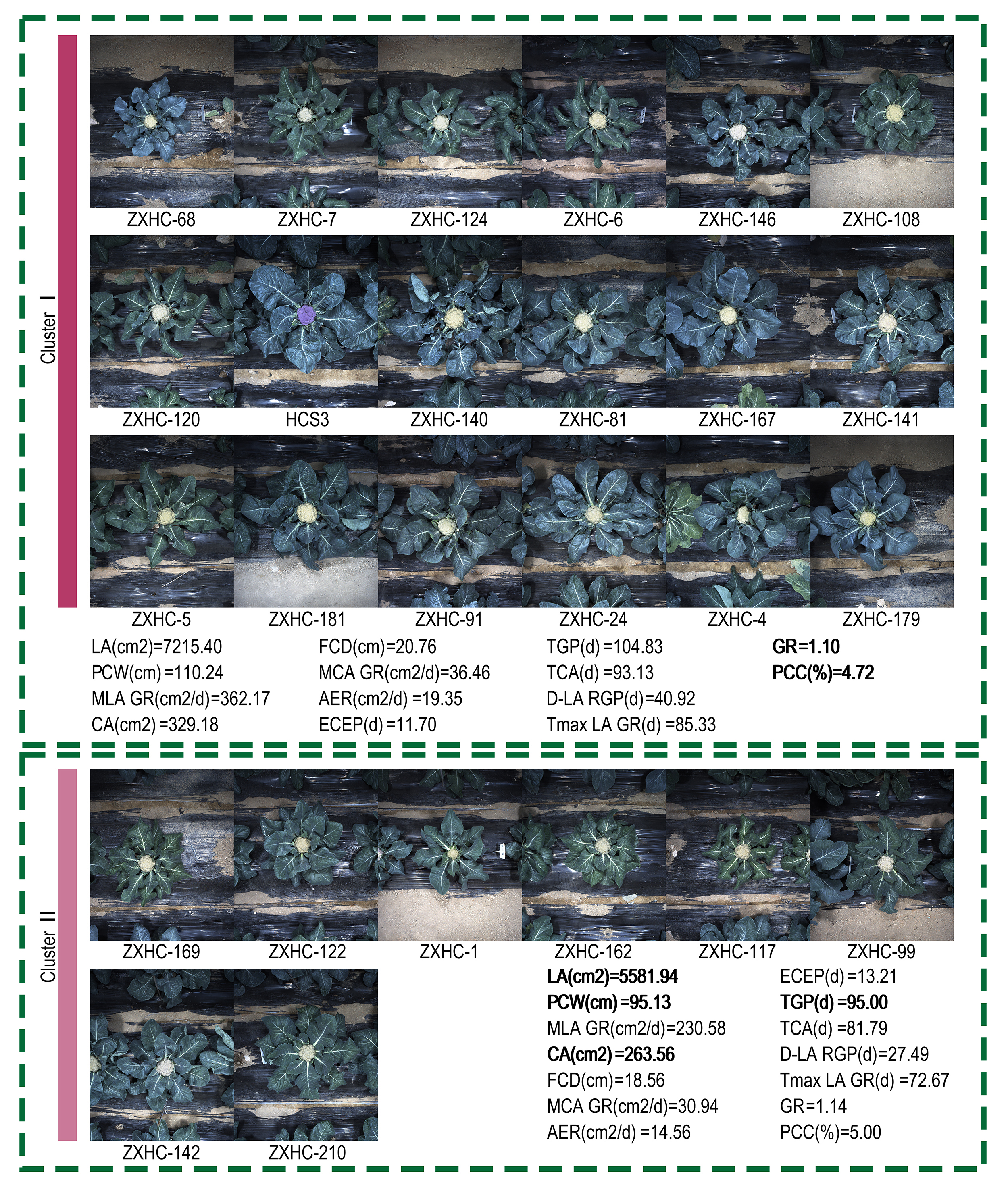

Supplement: Supplementary file 1 [file Image1.jpeg]

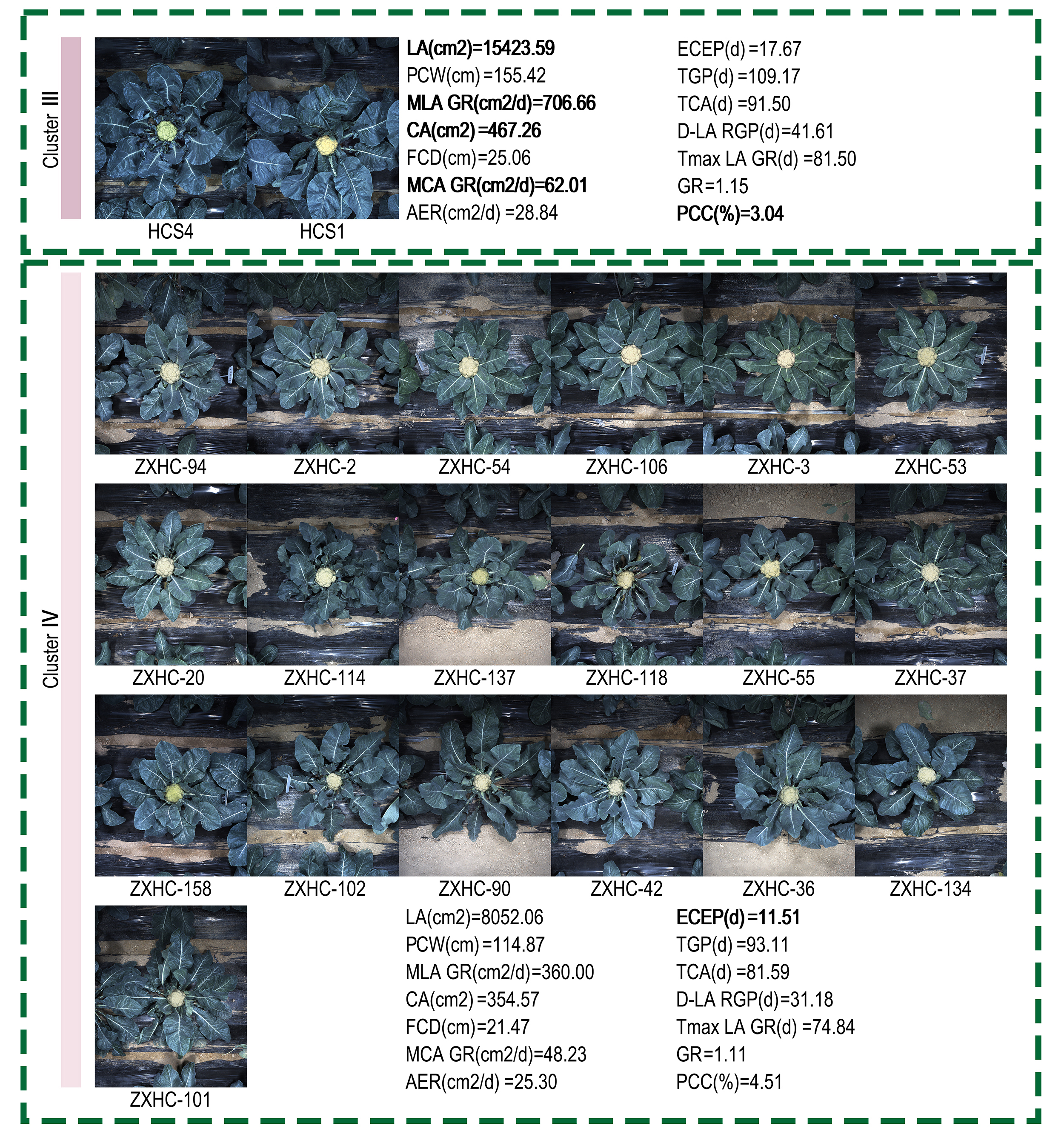

Supplement: Supplementary file 2 [file Image2.jpeg]
