# Supplementary material for: High-throughput phenotypic analysis of plant and curd growth dynamics during the whole growth period of cauliflower based on instance segmentation
Source: Front Plant Sci. 2026 May 13;17:1836813. doi: 10.3389/fpls.2026.1836813 (PMC13212518; doi:10.3389/fpls.2026.1836813)

### Supplementary Methods: Definitions of Evaluation Metrics

Each metric can be quantitatively described by the following formulas:

$P=\frac{\mathrm{TP}}{TP+FP}$ （1）

$R=\frac{\mathrm{TP}}{TP+FN}$ （2）

$\mathrm{AP}=\int_{0}^{1} P(R)dR$ （3）

$\mathrm{mAP}_{50}=\frac{1}{n_{c}}\int_{0}^{1} P(R)dR$ （4）

$\mathrm{mAP}_{50-95}=\mathrm{avg}(\mathrm{mAP}_{i}),i=50,55,...,95$ （5）

$Params=C_{\mathrm{in}}\times K^{2}\times C_{\mathrm{out}}$ （6）

$FLOPs=2\times H\times W\times\left( C_{\mathrm{in}}\cdot K^{2}+1 \right)\cdot C_{\mathrm{out}}$ （7）

$FPS=1/T\_inference$ （8）

In Equations (3), (4) and (5), AP represents the average precision obtained by integrating the area under the precision-recall curve; P(R) denotes the precision at a given recall level R; and dR is the integral variable corresponding to an infinitesimal change in recall. A higher average precision reflects better overall performance of the model in accurately detecting and segmenting cauliflower plants and curds. nc indicates the number of subcategories within the same object class.

In Equation (6), C_in_ is the number of input channels, C_out_ is the number of output channels, and K is the kernel size.

In Equation (7), H and W represent the height and width of the output feature map, respectively, and the constant term “1” accounts for the bias included in each convolution kernel.

In Equation (8), T_inference represents the average time required by the model to process a single frame of input data. This duration typically includes the entire pipeline of data preprocessing, model forward propagation (inference), and result post-processing.


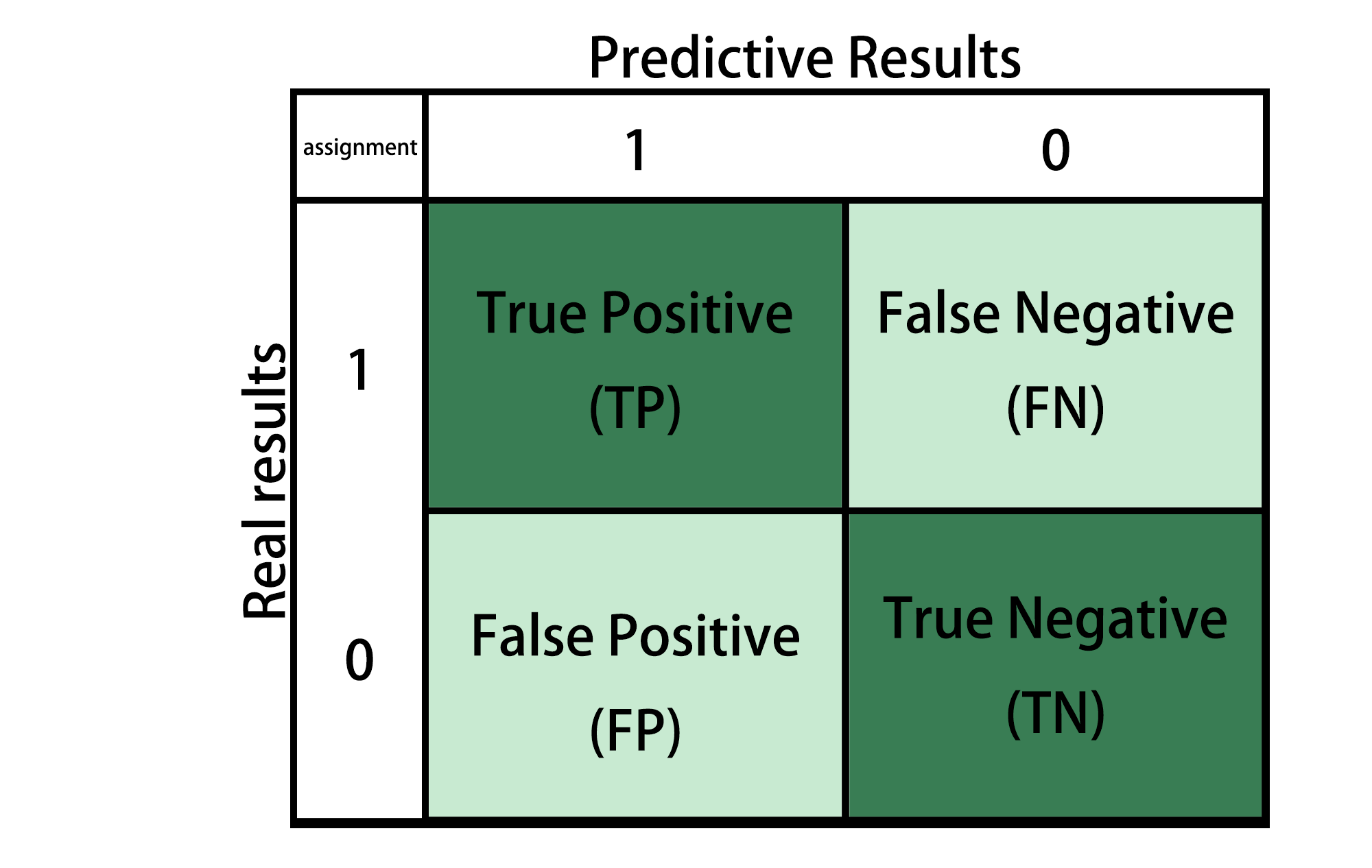

Supplement: Supplementary file 6 [file Table4.docx]
